# Atomic‐Scale Visualization and Quantification of Configurational Entropy in Relation to Thermal Conductivity: A Proof‐of‐Principle Study in *t*‐GeSb_2_Te_4_

**DOI:** 10.1002/advs.202002051

**Published:** 2021-02-08

**Authors:** Yongjin Chen, Bin Zhang, Yongsheng Zhang, Hong Wu, Kunling Peng, Hengquan Yang, Qing Zhang, Xiaopeng Liu, Yisheng Chai, Xu Lu, Guoyu Wang, Ze Zhang, Jian He, Xiaodong Han, Xiaoyuan Zhou

**Affiliations:** ^1^ College of Physics and Center for Quantum Materials and Devices Institute of Advanced Interdisciplinary Studies Chongqing University Chongqing 401331 P. R. China; ^2^ Beijing Key Laboratory and Institute of Microstructure and Property of Advanced Materials Beijing University of Technology Beijing 100124 P. R. China; ^3^ Center for High Pressure Science and Technology Advanced Research Beijing 100094 P. R. China; ^4^ Analytical and Testing Center Chongqing University Chongqing 401331 P. R. China; ^5^ Key Laboratory of Materials Physics Institute of Solid State Physics Chinese Academy of Sciences Hefei 230031 P. R. China; ^6^ Science Island Branch of Graduate School University of Science and Technology of China Hefei 230026 P. R. China; ^7^ Chongqing Institute of Green and Intelligent Technology Chinese Academy of Sciences Chongqing 400714 P. R. China; ^8^ University of Chinese Academy of Sciences Beijing 100044 P. R. China; ^9^ Center of Electron Microscopy and State Key Laboratory of Silicon Materials Department of Materials Science and Engineering Zhejiang University Hangzhou 310027 P. R. China; ^10^ Department of Physics and Astronomy Clemson University Clemson SC 29634‐0978 USA

**Keywords:** configurational entropy, single crystalline GeSb_2_Te_4_, thermal conductivity

## Abstract

It remains a daunting task to quantify the configurational entropy of a material from atom‐revolved electron microscopy images and correlate the results with the material's lattice thermal conductivity, which strides across statics, dynamics, and thermal transport of crystal lattice over orders of magnitudes in length and time. Here, a proof‐of‐principle study of atomic‐scale visualization and quantification of configurational entropy in relation to thermal conductivity in single crystalline trigonal GeSb_2_Te_4_ (aka *t*‐GeSb_2_Te_4_) with native atomic site disorder is reported. A concerted effort of large *t*‐GeSb_2_Te_4_ single crystal growth, in‐lab developed analysis procedure of atomic column intensity, the visualization and quantification of configurational entropy including corresponding modulation, and thermal transport measurements enable an entropic “bottom‐up” perspective to the lattice thermal conductivity of *t*‐GeSb_2_Te_4_. It is uncovered that the configurational entropy increases phonon scattering and reduces phonon mean free path as well as promotes anharmonicity, thereby giving rise to low lattice thermal conductivity and promising thermoelectric performance. The current study sheds lights on an atomic scale bottom‐up configurational entropy design in diverse regimes of structural and functional materials research and applications.

## Graphical abstract

A proof‐of‐principle study of atomic‐scale visualization and quantification of configurational entropy in relation to thermal conductivity in single crystalline trigonal GeSb_2_Te_4_ with native atomic site disorder is reported. The configurational entropy spontaneously intensifies phonon scattering and promotes anharmonicity, which leads to significantly reduced phonon mean free path, enabling an entropic “bottom‐up” perspective to the lattice thermal conductivity.

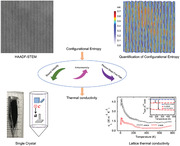

Entropy is ubiquitous and decisive in thermodynamics and thermal transport.^[^ ^1^, ^2^ ^]^ When a crystallographic site is competed by two or more elements, a site occupational disorder occurs. The configurational entropy (CE) gauges the level of site occupational disorder and thus governs the lattice thermal conductivity *κ*_L_.^[^ ^3^, ^4^, ^5^, ^6^ ^]^ In diverse research and application regimes of structural and functional materials, there has been a long pressing need for direct visualization of site disorder and quantification of CE in relation to the material's lattice thermal conductvitiy. However, from site disorder and configurational entropy to lattice thermal conductivity, it spans statics and dynamics of crystal lattice as well as the kinetics of thermal transport over orders of magnitude in spatial and temporal domain. One major barrier is concerned with the experimental method of quantifying the site disorder and configurational entropy with high spatial resolution.

To date, electron microscopy and X‐ray diffraction (XRD)^[^ ^7^, ^8^ ^]^ have been the two major methods for high resolution structural investigations. Either method has technical merits and disadvantages. Despite good data statistics and the capability of measuring atomic displacement parameters, the structural refinements of XRD data via Rietveld analysis yield an averaged crystal structure in the presence of site disorder, and there is an inherent structural ambiguity due to the missing phase angle. By contrast, the rapid progress of aberration‐corrected transmission electron microscopy (TEM) allows atom‐resolved investigation of site disorder and its spatial distribution.^[^ ^9^, ^10^, ^11^, ^12^ ^]^ But, a TEM image is largely a 2D static projection of a 3D vibrating lattice; the key information of depth profile and lattice dynamics are largely missing or averaged out. In addition, there is a dilemma between the statistics and high spatial resolution for electron microscopy study. It remains a daunting task to quantify the CE of a material from atom‐revolved electron microscopy images and correlate the results with the material's *κ*_L_, which strides across statics and dynamics of crystal lattice over orders of magnitudes in length and time. Overcoming the disadvantages of electron microscopy and making it a feasible way to quantify CE in relation to the lattice thermal conductivity is the main theme of this work.

Here, we conduct a proof‐of‐principle study by (*i*) developing a TEM protocol to deduce the depth profile of site occupation from intensity differential analysis of atomic columns, which has good statistics comparable with XRD, and an algorithm to quantify and map CE at atomic resolution. (*ii*) Growing large single crystals of trigonal GeSb_2_Te_4_ (crystal system: trigonal, space group $R\bar{3}m$, hereafter named *t*‐GST) to measure thermal and electrical transport properties along major crystallographic directions and over a wide temperature range. Compared to the metastable face centered cubic phase of GST (fcc, space group $Fm\bar{3}m$) and its amorphous counterpart, the *t*‐GST is a thermodynamically stable phase featured by coexistence of native cationic site disorder and long‐range order of crystal lattice.^[^ ^13^, ^14^, ^15^, ^16^, ^17^ ^]^ Fast reversible phase transitions can be attained among these phases by external stimuli.^[^ ^18^ ^]^ (*iii*) uncovering the relation between atomic‐scale CE and thermal conductivity in single crystalline *t‐*GST with native atomic site disorder by identifying anharmonicity based on first principles calculations, revising the mass and strain fluctuation terms in the Debye–Callaway model supplemented with CE, and measuring the heat capacity.

The high angle annular dark field (HAADF) scanning transmission electron microscopy (STEM), which is the primary technique used in this work, has elemental sensibility: the brightness is proportional to an exponential function of the atomic number Z.^[^ ^9^, ^12^ ^]^ **Figure** **1**a and **Figure** **2** (b) show the atomic‐resolved low magnification and enlarged HAADF‐STEM images along the $\left[11\bar{2}0\right]$ direction of the *t‐*GST sample, respectively. The HAADF‐STEM image of *t‐*GST clearly identifies the van der Waals gap and the septuplet substructure in the sequence of —Te1—GS2—Te2—GS1—Te2—GS2—Te1—, composed of bright anions and dark cations. Note that the CE is a scalar, which should not depend on the direction of observation. The choice of $\left[11\bar{2}0\right]$ zone axis is to minimize the blocking of atoms in the imaging direction, better extracting the intensities of atomic columns, characterizing the site occupational disorder of GS1 and GS2 layers, so as to quantify the CE. Different configurations may be obtained along different crystal orientation, but the outcome should not affect the CE. Specifically, Dr. Probe software^[^ ^19^ ^]^ was used to quantitatively simulate atomic scale HAADF‐STEM images of the GeSb_2_Te_4_ crystal along $\left[11\bar{2}0\right]$ orientation based on five atomic structure models with different thickness. The details and parameters for HAADF‐STEM image simulations can be found in the Section S3, Supporting Information. Figure 1c–e shows the comparison between the experimental and simulated HAADF results. As shown, the randomly selected and extracted experimental intensities of three septuple‐structures agree well with the average structure of disorder model (—Te—Ge_25_/Sb_75—_Te—Ge_50_/Sb_50_—Te—Ge_25_/Sb_75_—Te—).^[^ ^8^, ^15^ ^]^ The site occupancy of Ge/Sb are reconstructed by an in‐lab developed algorithm of nearest neighbor atomic column intensity differential analysis (IDA) (cf. the details and Figure S1–8 in the Supporting Information). The IDA method enables a coordinate encoding analysis of each atomic column, and the uncertainty of data analysis is as low as about ±4% (Figure S7, Supporting Information). The absolute intensity and normalized intensity maps for the atomic columns derived from Figure 1a are presented in Figure 1f and Figure 1g, respectively. The statistics of the normalized intensity of GS1 and GS2 cationic sublattice sites over 3000 cation atomic columns were presented on a quantitative map in Figure 1h. Ge/Sb ratios of each atomic column are shown in Figure 1i,j, and the corresponding statistics data present in Figure S6, Supporting Information. The GS1 layer possesses higher Ge fraction than the GS2 layer. The occupancy histograms shown in Figure 1k derived from about 3000 cationic columns obey a normal distribution with 49.9% ± 4% Ge in the GS1 layer and 26.9% ± 4% Ge in the two adjacent GS2 layers, in good agreement with the results of X‐ray diffraction.^[^ ^8^ ^]^ It is worth mentioning that owing to the IDA approach, the intensities collected from the individual atomic columns in the atomic‐resolved real space STEM image not only attain atomic resolution but also have good data statistics comparable to the X‐ray diffraction.

**Figure 1:**
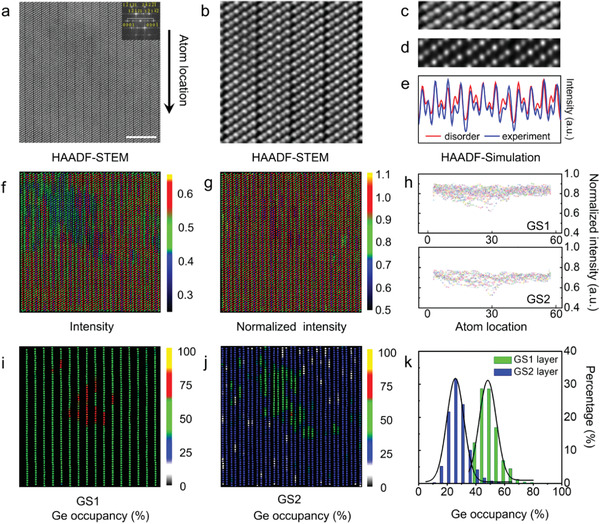
Atomic‐resolved atomic site occupational disorder in *t‐*GST. a) HAADF‐STEM image of layered structure along the [11$\bar{2}$0] direction, with the corresponding fast Fourier transform (FFT) pattern inset. The scale bar is 5 nm. b) A partial region of (a). c,d) The experimental and simulated HAADF‐STEM image （Te—Ge_25_/Sb_75_—Te—Ge_50_/Sb_50_—Te—Ge_25_/Sb_75_—Te—, at 40 nm thickness by Dr. Probe software), respectively. e) The intensity comparison of experiment and simulation on the same absolute intensity scale and thickness. f–g) The absolute intensity image and the normalized intensity image, respectively, derived from the spot intensity in HAADF image corresponding to (a). h) The statistics data of normalized intensity of GS1 and GS2 cationic sublattice sites over 3000 cation atomic columns; the atom location direction shown in (a). i,j) The concentration and disorder distribution of Ge atoms of individual GS1 and GS2 cationic sublattice sites, respectively. The mid‐value of color bar is set to be 50% (green), while the darker and brighter spots vary from 0 (gray) to 100% (yellow), roughly representing Ge‐poor to Ge‐rich columns. k) The Ge occupancy histogram of different layers over 3000 cation atomic columns.

**Figure 2:**
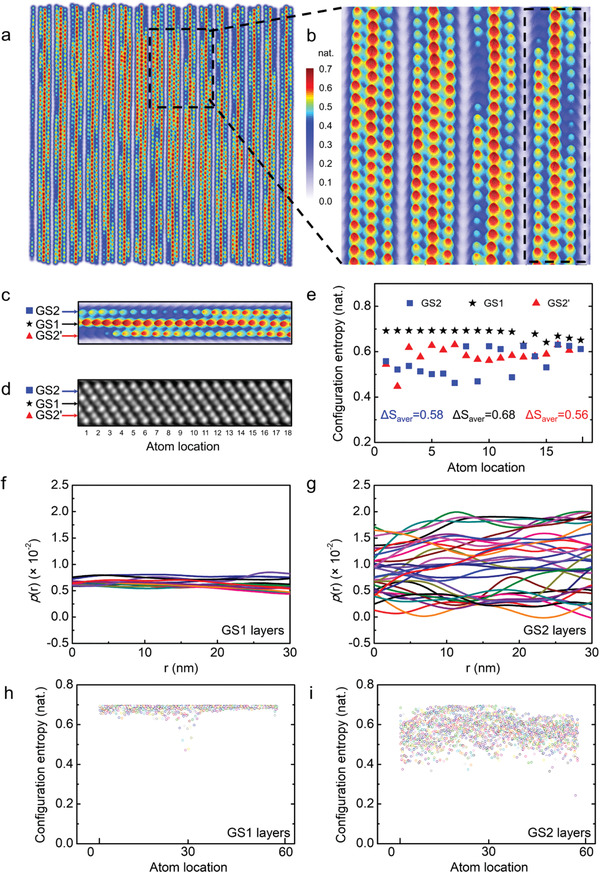
Configurational entropy mapping of *t‐*GST. a) The atomic‐resolved atomic column configurational entropy mapping of Figure 1a. b) An enlarged view of entropy mapping of a section of (a). c) The magnified region in (b). d) The corrsponding HAADF‐STEM image of (c). e) The modulation of atomic column configurational entropy correponding to (d); the *S_av_* of *t‐*GST cationic sublattice GS1, GS2, and GS2’ are 0.68, 0.56, and 0.58 nats, respectively. f–g) The curves of pair correlation function *p(*r) of GS1 and GS2 layers against distance *r*. h,i) The statistics data of atomic column CE of GS1 and GS2 cationic sublattice sites, respectively.

The IDA method is a “bottom‐up” method to derive the CE, while the XRD is a “top‐down” method to derive the CE, which are scientifically complementary. The precise data of atomic site occupational disorder derived above allow us to extract and quantify CE of *t‐*GST and reconstruct the atomic resolved images of CE for the first time. We define the CE of *i* ^th^ atomic column as Δ*S*_i_ based on the concept of entropy^[^ ^20^ ^]^ and expressed in terms of a discrete set of probabilities:

(1) $$\Delta S_{i}=\;-\sum_{j}^{n}f_{ij}\log_{b}f_{ij},\sum_{j}^{n}f_{\mathrm{ij}}=\;1\;$$

where *f*_ij_ is the mole content of the *j* ^th^ component of *i* ^th^ atomic column probability function, *b* is the base of the logarithm, and the value of *b* is Euler's number *e*; the units of entropy are the nats. (cf. Supporting Information for details) Entropy is extensive in nature, so the macroscopic CE is a sum over all the atomic columns. For two different atoms (that is Ge and Sb) within each atomic column, *f*_1_ and *f*_2_ are the fractional occupancies, where *f*_1_ +  *f*_2_ =  1. In this study, the values of *f*_1_ and *f*_2_ are experimentally determined by STEM studies and then used to verify the phonon scattering mechanism.

The CE of an atomic column is further simplified via Taylor expansions,

(2) $$\Delta S_{i}=-\left[f_{1}lnf_{1}+f_{2}lnf_{2}\right]\approx 2.5*f_{1}f_{2}$$

In the case that the Ge/Sb molar contents of atomic columns obey the normal distribution *ψ*(*μ*, *δ*) (Figure 1k). The average CE over all atomic columns is

(3) $$\;\Delta S_{\mathrm{av}}=2.5\left[\mu \left(1-\mu \right)-\delta^{2}\right],0<f<1$$

where *μ* and *δ* are the expectation and variance of the normal distribution, respectively.

Figure 2a presents the atomic‐resolved mapping of CE for *t‐*GST, in which the degree of cationic site disorder is directly visualized and quantified. To simplify the analysis yet without altering the main conclusion, the anionic antisitic defects are not considered as its percentage is <3% (cf. Table S3, Supporting Information), comparable to the uncertainty of our data analysis. Figure 2b displays an enlarged view of CE mapping of a selected area in Figure 2a. A framed slice of HAADF image (18 × 3 GS1 and GS2 atomic columns), the corresponding CE image, and the value of CE for each atomic column are shown in Figure 2c–e, respectively. In contrast to the HAADF image, it is apparent that the CE values are largely fluctuated, especially within the GS2 atomic layers. The pair correlation function *p(*r) can be used to describe the modulation period.^[^ ^21^ ^]^ Figure 2f,g show the curves of *p(*r) of GS1 and GS2 layers against distance *r*, where *r* is the spacing between any two CE peaks in a particular atomic layer. The smooth curves of GS1 layers reveal a high frequency CE modulation (1/3 Å^−1^) and the short‐range order of Ge/Sb, and the characteristic length of modulation of atomic column CE of GS1 layer is estimated to be close to the interatomic spacing ∼3 Å. By contrast, a sharp distinctive feature occurs in the GS2 layers, and the characteristic length of GS2 layer is found to be at about 9 Å with a low frequency CE modulation (1/9 Å^−1^). To be more general, the data containing 60 × 50 (layers) atom columns were analyzed. The CE of GS1 layers and GS2 layers are shown in Figure 2h,i, reflecting the modulation feature of CE. High CE values beyond 0.6 nats, which correspond to an atomic ratio of Ge/Sb ≈ 1:1, were found for most GS1 layers. In contrast, the CE values of GS2 layers are mostly between 0.4 and 0.69 nats. The *S*_av_ of *t‐*GST cationic sublattice is found to be 0.61 nats. The modulation of atomic‐column CE and the distinct entropic behavior in GS1 and GS2 layers are uncovered, which will give rise to the strain effects and contribute to the anharmonicity that will be demonstrated in the following sections.

Relating to the native cationic site disorder and CE, the specific heat and thermal conductivity of single crystalline *t‐*GST along major crystallographic axes from 5 to 773 K are investigated. **Figure** **3**a displays the as‐grown large size *t‐*GST single crystal cleaved along the (0001) plane and the schematic of transport measurement directions. Figure 3b presents the temperature dependent isobaric specific heat *C*_p_, showing the lattice dynamics of *t‐*GST. At high temperatures, the *C*_p_ goes above the classic Dulong–Petit limit by about 12%, a sign of anharmonicity. A fairly large anisotropy of the total thermal conductivity (*κ*_tot_) and *κ*_L_ between *a‐*axis (parallel to the cleavage planes) and *c‐*axis (perpendicular to the cleavage planes) is shown in Figure 3c, despite similar temperature dependence. The conspicuous rising tail of *κ*_tot_ observed in *c‐*axis above 623 K is ascribed to the bipolar effect in view of the narrow band gap (0.14 eV) of *t‐*GST. In light of the thermoelectric promise of PCMs,^[^ ^18^ ^]^ the thermoelectric performance of *t‐*GeSb_2_Te_4_ was also evaluated along major crystallographic axes over a wide temperature range, a figure of merit ≈0.8 was attained at 723 K along the *c*‐axis (Figure S28, Supporting Information). Between 30 and 300 K, the *κ*_tot_ along *c‐*axis slowly decreases with decreasing temperature, before drastically increasing with further decreasing temperature, forming a maximum locating at ~ 18 K. The peak is a feature of crystalline material due to the competition between the specific heat and Umklapp scattering. The peak position is usually about 10–20% of the Debye temperature, in good accordance with the data presented in Figure 3b. The peak values of *κ*_tot_ are about 2.09 W m^−1^ K^−1^ along *c‐*axis and 5.85 W m^−1^ K^−1^ along *a‐*axis. A *κ*_L_ value as low as 0.40 W m^−1^ K^−1^ along *c‐*axis at 300 K is attained in Figure 3d. By contrast, *κ*_L_ along *a‐*axis at 300 K is 1.4 W m^−1^ K^−1^. Though it is not straightforward, a comparison by collecting data of thermal conductivity from literature of two related materials without site occupation disordering Sb_2_Te_3_ and GeTe was summarized in Figures S26 and Figure S27, Supporting Information, respectively. The *κ*_L_ of *t‐*GST at 300 K is about only one‐fifth of that of polycrystalline GeTe, and 66% of that of single crystal Sb_2_Te_3_. These results give additional information and suggest that CE significantly contributes to lowering thermal conductivity of a crystalline material.

**Figure 3:**
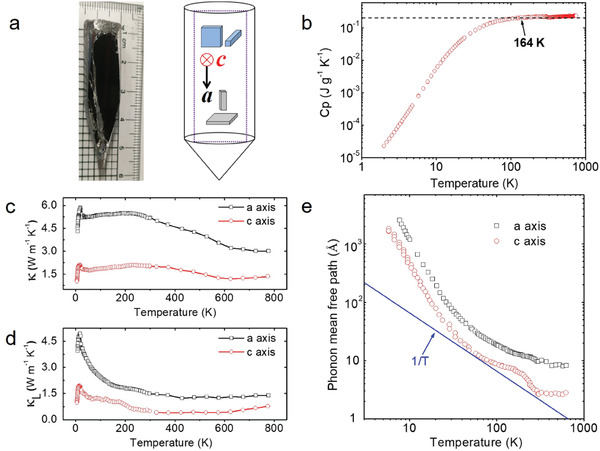
The optical image, specific heat, thermal conductivity, and phonon mean free path of single crystalline *t‐*GST. a) A typical crystal cleaved along the (0001) plane and the cartoon illustration of samples cutting along two major crystallographic directions (*a* and *c*) for transport measurements. b) The specific heat capacity. c) Temperature dependence of total thermal conductivity (*κ*) from 5 K to 773K. d) Lattice thermal conductivity (*κ*_L_). e) The phonon mean free path (*l*).

A rule of thumb in the thermal transport of a crystalline material is that the average wavelength of heat‐carrying phonons is lattice constant × (temperature/Debye temperature).^[^ ^22^ ^]^ For *t‐*GST, the Debye temperature is 164 K estimated from the fitting to the low temperature data of *C*_p_ (Figure 3b). Therefore, the average wavelength of heat carrying phonons of *t‐*GST is reduced to the order of lattice constant or less in the vicinity and above room temperature. In a phenomenological kinetics manner, *κ*_L _ is expressed as $\;\kappa_{L}=\frac{1}{3}\;C_{V}\vartheta l$, where *C_V_*, ϑ, and *l* are the isochoric specific heat, the average sound velocity, and phonon mean free path (PMFP), respectively. The CE is embodied in the term *l* as the site disorder shortens the PMFP and in turn suppress *κ*_L_. Given a weak temperature dependence of ϑ (Table S5, Supporting Information), specific heat and sound velocity were used to derive PMFP shown in Figure 3e. The PMFP along *a‐*axis and *c‐*axis are found to decrease with increasing temperature, consistent with a crystalline material, and largely follow a power law behavior but the exponents are notably deviated from 1/T. The PMFP along *c‐*axis (*a‐*axis) at 300 K is about 3.0 Å (9.6 Å), which is on the same order of the characteristic length of modulation of atomic column CE derived in entropy study (Figure 2). The sub‐nano scale modulation of atomic column CE will facilitate phonon scattering because the characteristic length of CE modulation and PMFP are comparable. The intrinsically low *κ*_L_ and significantly reduced PMFP result from strong phonon–cationic disorder scattering in the single crystalline *t‐*GST, a testimony to the important role of CE.

We further conduct first principles calculations to give insights into the anharmonicity of *t‐*GST by comparing the Grüneisen parameter *γ* of two phases of *t‐*GST, that is, in low and high CE state. In parallel with the phonon mean free path in spatial domain, anharmonicity suppresses *κ*_L_ via shortening the phonon lifetime in temporal domain.^[^ ^23^ ^]^ The anharmonicity is characterized by the Grüneisen parameter *γ*, which can be estimated by the computationally feasible elastic properties.^[^ ^24^ ^]^ Unlike the origin of CE in extrinsically doped materials, the CE of *t*‐GST stems from its “native” site occupational disorder. The most straightforward way to showcase the effect of CE is the theoretical calculations of the same crystal structure (including the van der Waals gaps) but with different levels of site occupational disorder. Two structural models are built as shown in **Figure** **4**a,b, namely, *(i)* a hypothetical *t‐*GST without Ge/Sb site occupational disorder (pristine order) of *t‐*GST, *(ii)* the actual high CE state (high‐ΔS) of *t‐*GST (based on STEM observation in Figures 1 and 2). The high entropy *t‐*GST structure is simulated using a Special Quasi‐random Structure (SQS)^[^ ^25^ ^]^ (cf. Experimental Section and Supporting Information). The electron localization function in Figure 4a,b gives the extent of localized electrons and reflects the relative strength of a chemical bond between two atoms. As shown, the localized electrons only exist on the Te1 and Sb sites in pristine ordered GST, while they appear in the Te2 and GS1 sites in disordered *t‐*GST. The coulomb repulsion between the localized electrons weakens the chemical bond, and modulates the bond length and bond angle (cf. Figure S18, Supporting Information). The high‐ΔS GST has lower sound velocity (1406 m s−1) and larger Grüneisen parameter (~3.2) than the pristine ordered GST (cf. Table S6, Supporting Information). Hence, the CE in *t‐*GST turns out to be an indicator of both site disorder (affecting phonon mean free path) and anharmonicity (affecting phonon life time). From previous reports^[^ ^26^, ^27^ ^]^ and our density functional theory calculations, the ranking of anharmonicity from higher to lower is *fcc*‐GST, high‐ΔS *t‐*GST, pristine ordered *t‐*GST, and amorphous GST. Note that the native site occupational disorder introduces CE that helps to stabilize the *t‐*GST phase. As shown in Figure 4c, we compare two phases of *t‐*GST by theoretically calculating their Gibbs free energy. The Gibbs free energy differences in diverse configurations are found to be fairly small, on the order of several meV per atom. Nonetheless, the Gibbs free energy of pristine ordered GST is the lowest at *T* = 0 K. At elevated temperatures, it is the configurational entropy that stabilizes the high entropy phase of *t‐*GST and deduces the absence of pristine–ordered GST in practice.

**Figure 4:**
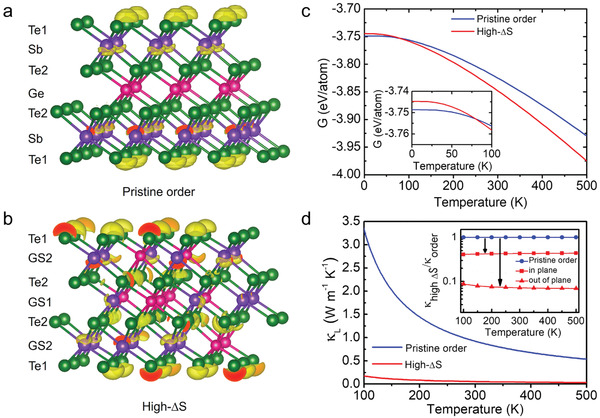
The calculated lattice thermal conductivity and thermodynamics stability of pristine ordered and high entropy disordered *t‐*GST. a,b) The structure models and the electron localization function (ELF) of GST in the pristine ordered and high entropy disordered system, which reflect the relative strength of a chemical bond. The isosurface threshold value is 0.86, Te (cyan), Sb (purple), and Ge(pink). c) The Gibbs free energy of two *t‐*GST phases with different configurational entropy. d) The calculated lattice thermal conductivity. The inset shows the configurational entropy's contribution to the reduction of lattice thermal conductivity. The notations “in” and “out” denote the ratio of *κ*_high−Δ_ *_S_/κ*_order_ in high‐ΔS disordered *t‐*GST along the in‐plane and out‐of‐plane directions, respectively.

Finally, we evaluate the contribution of CE to the thermal conductivity from the theoretical considerations and experimental data obtained above. The Allan–Feldman model,^[^ ^28^ ^]^ normal‐mode decomposition,^[^ ^29^ ^]^ and virtual crystal approximation^[^ ^30^ ^]^ have been used to estimate the effect of disorder on the phonon life time and/or the lattice thermal conductivity, but the effects of anharmonicity or local geometry distortion (also known as the strain effect) are left out. Here, we revised the mass fluctuation scattering term and strain fluctuation term in the Debye–Callaway model^[^ ^31^, ^32^, ^33^ ^]^ with CE. The scattering factors/parameters derived from the IDA of TEM data are plugged into the classic Debye–Callaway^[^ ^31^, ^32^, ^33^ ^]^ and Klemens model^[^ ^34^ ^]^ to quantify the impact of CE on the lattice thermal conductivity in major crystallographic axes. Specifically, we revised the mass fluctuation scattering term and strain fluctuation term in the Debye–Callaway model supplemented with CE (For details, see Experimental Section and Supporting Information).

(4) $$\begin{array}{c} \Gamma_{\exp}=\frac{\Delta S}{2.5}\;\frac{\sum_{i\;=\;1}^{n}c_{i}{\left(\frac{\bar{M_{i}}}{\bar{\bar{M}}}\right)}^{2}{\left(\frac{M_{i}^{1}-M_{i}^{2}}{\bar{M_{i}}}\right)}^{2}+\;\sum_{i\;=\;1}^{n}c_{i}{\left(\frac{\bar{M_{i}}}{\bar{\bar{M}}}\right)}^{2}\varepsilon {\left(\frac{r_{i}^{1}-r_{i}^{2}}{\bar{r_{i}}}\right)}^{2}}{\left(\sum_{i\;=\;1}^{n}c_{i}\right)} \end{array}$$

In the case of *t‐*GST, *n* = 2 because of 2 sublattices. As above, we estimate the value of Γ_*exp*_ to be about 0.27, and the scattering parameter is related to not only the atomic mass and size difference, but also the CE. Based on the theory of Klemens,^[^ ^34^ ^]^ the ratio of $\kappa_{L}^{\Delta S}$ of the high‐ΔS *t‐*GST to that of pristine ordered *t‐*GST, $\kappa_{L}^{p}$, can be expressed as

(5) $$\frac{\kappa_{L}^{\Delta S}}{\kappa_{L}^{p}}=\frac{\tan^{-1}u}{u},\;\;u^{2}=\frac{\pi^{2}\theta_{D}V}{h\vartheta_{m}^{2}}\kappa_{L}^{p}\;\Gamma_{exp}$$

where *u*, *V*, *h*, and ϑ_*m*_ are the disorder scaling parameter, the average volume/atom, the Planck constant, and the average sound velocity, respectively. Thus, the disorder scaling parameter *u* and the ratio of $\frac{\kappa_{L}^{\Delta S}}{\kappa_{L}^{p}}$ can be derived by combining Equations (4) and (5), where the calculated $\kappa_{L}^{p}$ of pristine ordered *t‐*GST was used, yielding *u =* 1.63 and 2.7 at 200 K and the ratio of $\frac{\kappa_{L}^{\Delta S}}{\kappa_{L}^{p}}=\;$62% and 45% for the *a‐*axis and *c‐*axis, respectively.

The Debye–Callaway model^[^ ^31^, ^32^, ^33^ ^]^ was also applied to simulate the averaged and directional lattice thermal conductivities based on the data of density–functional theory calculations, and the results are presented in Figure 4d; and Figure S19, Supporting Information. It is found that the lattice thermal conductivity of high‐ΔS *t‐*GST is low and of weak temperature dependence, and strain fluctuations dominate the phonon scattering in *t‐*GST, though mass fluctuations cannot be neglected (Figure S19, Supporting Information). The van‐der‐Waals gap plays a key role in restricting the thermal transport along the *c*‐axis, yielding fairly large anisotropy of lattice thermal conductivity. Nonetheless, this is not at odds with the results of CE. Toward lowering lattice thermal conductivity, the presence of van der Waals gap and CE are two key contributions. The inset in Figure 4d shows the CE's contribution to lower lattice thermal conductivity; the CE reduced the lattice thermal conductivity by more than 50% regardless along the in‐plane or out‐of‐plane direction. The results of experimental data are compared with those of first principles calculations. As shown in Figure 4d, the results match well along *a‐*axis, while the match along the *c*‐axis is less satisfactory, which can be attributed to the presence of van der Waals gap. The contribution of van der Waals gap is considered in the first principle calculations but not in the Debye–Callaway and Klemens model. These results point toward the CE contributing to phonon scattering and low lattice thermal conductivity.

In summary, the configurational entropy provides a general (thermodynamic) perspective to the phonon scattering, that is, a statistical index beyond the specific scattering mechanism. The atomic resolved configurational entropy is developed utilizing our newly developed technique of column intensity differential analysis. The successful growth of large size *t‐*GST single crystals with inherent site disorder enabled us to address the profound correlation across atomic scale site occupation disorder, sub‐nano scale CE modulation, and macroscale lattice thermal conductivity. We invoked atomic scale quantified and visualized CE to bridge “local” feature (the CE from one atomic column to thousands of atomic columns) and “global” transport properties (heat transport at the scale of mm or cm). These results may provide fundamental information and pave a route on the design of configurational‐entropy enabled functional and structural materials.

## Experimental Section

##### Sample Preparation and Characterization

Pure Ge (99.999%), Sb (99.999%), and Te (99.999%) were weighed according to the stoichiometry of Ge_1_Sb_2_Te_4_ and sealed into the cone‐shaped quartz tubes under ~2 × 10^−4^ Pa, subsequently placed in another quartz tube. The tubes were slowly heated up to 1273 K over 20 h and soaked at that temperature for 20 h. Then the tubes were cooled to 973 K as they were lowered at a rate of 1.3 mm h^−1^. The GeSb_2_Te_4_ single crystal ingots with the diameter of 14 mm and the height of 55 mm were obtained; the crystal structure and directions were determined using scanning electron microscope with electron back scattered diffraction system (Quanta 650 F, FEI) and advance diffraction system (Bruker D8, Cu K*_α_* radiation with *λ* = 0.15 418 nm). The differential thermal analysis (DTA) measurement showed that GST started to melt at 889 K. Temperature dependent XRD measurement was performed using PANalytical Empryean with Cu K*_α_* radiation. The ingots were cut into ~8.5 mm × 2 mm × 2 mm bars for electrical property measurements, which were performed on a commercial system (LSR‐3, Linseis) under a static helium atmosphere. The temperature dependent Hall coefficient (*R*_H_) was measured using a home‐made apparatus. The square‐shaped samples with dimensions ≈6 mm × 6 mm × 1.5 mm were also prepared for the thermal diffusivity (*D*) measurements (LFA 457, Netzsch). The heat capacity (*C*_p_) was obtained through differential scanning calorimetry (DSC 404 F3, Netzsch). The thermal conductivity (*κ*) was calculated using *κ*  =  *ρ* *DC*_p_, where *ρ* is the density measured by the Archimedes method on a commercial instrument (BR‐120N, Beyongtest). The lattice thermal conductivity was estimated by subtracting the electronic thermal conductivity *κ*_e_ (calculating based on the Wiedemann–Franz law) from the total thermal conductivity. The estimated errors in electrical resistivity, Seebeck coefficient, and thermal conductivity were about 3%, 5%, and 7%, respectively; and the overall uncertainty of *zT* at around 15% was estimated and error bars were displayed in relevant figures. Transport property measurement between 5 and 300 K was performed with a Quantum Design Physical Property Measurement System (PPMS, DynaCool‐9T) using Thermal Transport Option (TTO).

##### Scanning Transmission Electron Microscopy (STEM)

Cross‐sectional TEM specimens were prepared by a combination of Ga focus ion beam (FIB) and Ar low‐energy ion beam milling, with the final specimen thickness being 40 ± 5 nm (as measured by electron energy loss spectroscopy). The STEM images were performed at 300 kV on a probe‐corrected FEI Titan G2 60–300 microscope with a super energy dispersive X‐ray system. A probe forming aperture of 25 mrad for imaging of GST was used in the experiments. The images in this work were acquired with a HAADF detector using annular ranges of 80–200 mrad. The high order aberrations were reduced to values close to zero; the first order aberrations (A1 and C1) to values below 0.5 nm, and second order aberrations (A2 and B2) to values in the range of 5 nm. Beam currents were limited to ~80 pA. In order to reduce the influence of the electron beam on the possible randomization of Ge and Sb atomic species and ensure the good signal‐to‐noise ratio for IDA analysis, dwell times per pixel of 15 µs for 1 k × 1 k image size and 6 µs for 2 k × 2 k image size were used.

For the “nearest” neighbor atomic column intensity differential analysis (IDA), see Section S1, Supporting Information.

For HAADF‐STEM image simulations, see Section S3, Supporting Information.

##### Density Function Theory Calculations

Density‐functional theory calculations were performed using the Vienna Ab initio Simulation Package (VASP),^[^ ^35^ ^]^ which employs the projector augmented‐wave (PAW)^[^ ^36^, ^37^ ^]^ scheme and the generalized gradient approximation of Perdew, Burke, and Ernzerhof (GGA‐PBE)^[^ ^38^ ^]^ for the electronic exchange‐correlation functional. The energy cutoff for the plane‐wave expansion was 500 eV. The Brillouin zones were sampled by Monkhorst–Pack^[^ ^39^ ^]^ grids with a roughly constant *k*‐point density of 30 Å^3^ in all calculations. The crystal geometry was fully relaxed until the force components of each atom were below 0.01 eV Å^−1^. Here, two systems were compared: (i) the pristine ordered GST: Ge ions occupied the GS1 layer while Sb ions occupied the GS2 layer. The other system was disordered GST; there was a cross‐layer site occupational disorder, sometimes called “self‐solid‐solution”. The disordered GST was simulated using a Special Quasi‐random Structure (SQS), which satisfies the mixing ratios of Ge/Sb. Ge:Sb = 1:1 at the GS1 layer, and Ge:Sb = 1:3 at the GS2 layer, which is a high entropy state.

The elastic constants (C or C_ij_) are calculated from the strain‐stress relationship:^[^ ^40^ ^]^ *σ*_s_ =  *C* · *ε*, where *σ*_s_, *C*, and *ε* are the engineering stress vector, the stiffness matrix, and the strain vectors, respectively. According to the Voigt–Reuss–Hill (VRH) theory in a macroscopic system,^[^ ^41^ ^]^ the corresponding elastic properties, such as the bulk modulus *B* and shear modulus *G*, can be evaluated from the elastic constants.

##### Lattice Thermal Conductivity Evaluations

The Debye–Callaway model was employed to simulate the lattice thermal conductivity, which is expressed as:^[^ ^31^, ^33^ ^]^

(6) $$\kappa_{L}=\frac{k_{B}}{2\pi^{2}v}\;{\left(\frac{k_{B}T}{\hbar }\right)}^{3}\left[\int_{0}^{\Theta_{D}}\tau_{C}\frac{x^{4}e^{x}}{{\left(e^{x}-1\right)}^{2}}dx+\frac{{\left(\int_{0}^{\frac{\Theta_{D}}{T}}\frac{\tau_{C}x^{4}e^{x}}{\tau_{N}{\left(e^{x}-1\right)}^{2}}dx\right)}^{2}}{\int_{0}^{\Theta_{D}}\frac{\tau_{C}x^{4}e^{x}}{\tau_{N}\tau_{U}{\left(e^{x}-1\right)}^{2}}dx}\right]$$

where *k*_B_ is the Boltzmann constant, *ħ* is the Plank constant, *T* is the absolute temperature, *τ*_C_, *τ*_U_, and *τ*_N_ are the relaxation time, *x* = ℏ*ω*/*k*_B_T, *ω* is the phonon frequency, and *ν* is the acoustic phonon velocity. Using the elastic properties (bulk modulus *B* and Shear modulus *G*, Table S7), it is possible to estimate the phonon velocity (*v*) and Debye temperature (Θ_D_) as,^[^ ^24^ ^]^

(7) $$\nu ={\left[\frac{1}{3}\left(\frac{1}{\nu_{L}^{3}}+\frac{2}{\nu_{S}^{3}}\right)\right]}^{-\frac{1}{3}}\;,\nu_{L}=\sqrt{\frac{B+4/3G}{\rho }}\;,\nu_{S}=\sqrt{\frac{G}{\rho }}\;$$

(8) $$\Theta_{D}=\frac{\hbar }{k_{B}}\;{\left(\frac{3m}{4\pi }\right)}^{\frac{1}{3}}\nu n^{-\frac{1}{3}}$$

where *v*_L_ and v_S_ are the longitude and shear phonon velocities, and *m* and *n* are the number of atoms per volume and the number of atoms in the cell.

To simulate the lattice thermal conductivity, the phonon relaxation times must be known. For a disorder single crystalline material, the dominated phonon scattering processes are the Umklapp phonon–phonon scattering (*U*) and the Normal phonon–phonon scattering (*N*). Therefore, the relaxation time *τ*_C_ includes two contributions, $\tau_{C}^{-1}=\tau_{U}^{-1}\;+\tau_{N}^{-1}$. The relaxation time associated with the U scattering could be represented as:^[^ ^31^, ^42^, ^43^ ^]^

(9) $$\tau_{U}^{-1}=\frac{k_{B}^{2}\gamma^{2}}{\hbar M\nu^{2}\Theta_{D}}\;x^{2}T^{3}e^{-\Theta_{D}/3T}$$

where *M* and *γ* are the average mass per atom and the Grüneisen parameter, respectively. The Grüneisen parameter characterized the phonon anharmonicity, which normally requires time‐consuming first‐principles quasi‐harmonic phonon calculations.

Alternatively, a computationally efficient methodology was adopted to estimate the Grüneisen parameter based on elastic properties:^[^ ^24^ ^]^

(10) $$\begin{array}{ccc} \gamma & = & \sqrt{\left[\gamma_{L}^{2}+2\gamma_{S}^{2}\right]/3};\;\;\gamma_{L}=-\frac{1}{2}\frac{V}{B+\frac{4}{3}G}\frac{\partial \left(B+\frac{4}{3}G\right)}{\partial V}-\frac{1}{6};\\ \gamma_{S} & = & -\frac{1}{2}\frac{V}{G}\frac{\partial G}{\partial V}-\frac{1}{6} \end{array}$$

For the N scattering, the relaxation time is written as:^[^ ^31^ ^]^

(11) $$\tau_{N}^{-1}=\frac{k_{B}^{5}\gamma^{2}V}{2M\hbar^{4}\nu^{5}}\;\left(2x+x^{2}\right)T^{5}$$

The classic Debye–Callaway model correlates the macroscopic lattice thermal conductivity with the microscopic atomic mass difference and radius difference. The site disorder induced scattering parameter Γ is given by^[^ ^44^ ^]^

(12) $$\Gamma \;=\Gamma_{M}\;+\Gamma_{S}$$

where Γ_M_ and Γ_S_ are the scattering parameters due to mass fluctuations and strain field fluctuations, respectively. Given the different atomic mass and radii of Ge/Sb, the cationic‐site occupational disorder induced by mass fluctuations and strain field fluctuations in *t‐*GST can be rewritten as^[^ ^44^ ^]^

(13) $$\Gamma_{M}=\frac{\sum_{i=1}^{n}c_{i}{\left(\frac{\bar{M_{i}}}{\bar{\bar{M}}}\right)}^{2}f_{i}^{1}f_{i}^{2}{\left(\frac{M_{i}^{1}-M_{i}^{2}}{\bar{M_{i}}}\right)}^{2}}{\left(\sum_{i=1}^{n}c_{i}\right)}\;$$

(14) $$\Gamma_{S}=\frac{\sum_{i=1}^{n}c_{i}{\left(\frac{\bar{M_{i}}}{\bar{\bar{M}}}\right)}^{2}f_{i}^{1}f_{i}^{2}\varepsilon {\left(\frac{r_{i}^{1}-r_{i}^{2}}{\bar{r_{i}}}\right)}^{2}}{\left(\sum_{i=1}^{n}c_{i}\right)}$$

Where $M_{i}^{1}$, $M_{i}^{2},$ and $r_{i}^{1}$, $r_{i}^{2}\;$are the atomic masses and radii of *i_t_*_h_ sublattice (*i* = 1,2, GS1 and GS2 sublayers), $f_{i}^{1}$and $f_{i}^{2}$ are the mole content of Ge and Sb. $\bar{M}$ and $\bar{r}$ are the average mass and average atomic radius, the *c*_i_ are the relative degeneracies of the respective sites of Ge/Sb. $\bar{\bar{M}}$ is the the average mass. *ε* is a parameter of a function of the Grüneisen parameter *γ*. Hence, the disorder scattering parameter Γ_M_, Γ_S_, and Γ are expressed as,

(15) $$\Gamma_{M}=\frac{\sum_{i=1}^{n}c_{i}{\left(\frac{\bar{M_{i}}}{\bar{\bar{M}}}\right)}^{2}\frac{\Delta S}{2.5}{\left(\frac{M_{i}^{1}-M_{i}^{2}}{\bar{M_{i}}}\right)}^{2}}{\left(\sum_{i=1}^{n}c_{i}\right)}$$

(16) $$\Gamma_{S}=\frac{\sum_{i=1}^{n}c_{i}{\left(\frac{\bar{M_{i}}}{\bar{\bar{M}}}\right)}^{2}\frac{\Delta S}{2.5}\varepsilon {\left(\frac{r_{i}^{1}-r_{i}^{2}}{\bar{r_{i}}}\right)}^{2}}{\left(\sum_{i=1}^{n}c_{i}\right)}\;$$

(17) $$\Gamma \;=\frac{\Delta S}{2.5}\;\frac{\sum_{i\;=\;1}^{n}c_{i}{\left(\frac{\bar{M_{i}}}{\bar{\bar{M}}}\right)}^{2}{\left(\frac{M_{i}^{1}-M_{i}^{2}}{\bar{M_{i}}}\right)}^{2}+\;\sum_{i\;=\;1}^{n}c_{i}{\left(\frac{\bar{M_{i}}}{\bar{\bar{M}}}\right)}^{2}\varepsilon {\left(\frac{r_{i}^{1}-r_{i}^{2}}{\bar{r_{i}}}\right)}^{2}}{\left(\sum_{i\;=\;1}^{n}c_{i}\right)}$$

All tested data were carried out and plotted with Wolfram Mathematica (V9.0, Wolfram Research), Matrix Laboratory (V8.2, MathWorks), and OriginPro (V9.0, OriginLab Corp.). For bar diagrams, the data were presented as mean ± SD (standard deviation).

## Conflict of Interest

The authors declare no conflict of interest.

## Supporting information

Supporting Information

## Data Availability

Research data are not shared.
