# Supplementary material for: Atomic‐Scale Visualization and Quantification of Configurational Entropy in Relation to Thermal Conductivity: A Proof‐of‐Principle Study in t‐GeSb2Te4
Source: Adv Sci (Weinh). 2021 Feb 8;8(8):2002051. doi: 10.1002/advs.202002051 (PMC8061353; doi:10.1002/advs.202002051)
Supplement: Supplementary file 1 — Supporting Information [file ADVS-8-2002051-s001.pdf]

WILEY-VCH

© 2020 Wiley-VCH GmbH

ADVANCED SCIENCE
Open Access

Supporting Information

for *Adv. Sci.*, DOI: 10.1002/advs.202002051

Atomic-Scale Visualization and Quantification of Configurational Entropy in Relation to Thermal Conductivity: A *Proof-of-principle* Study in *t*-$GeSb_2Te_4$

Yongjin Chen, Bin Zhang, Yongsheng Zhang, Hong Wu, Kunling Peng, Hengquan Yang, Qing Zhang, Xiaopeng Liu, Yisheng Chai, Xu Lu, Guoyu Wang, Ze Zhang, Jian He, Xiaodong Han, and Xiaoyuan Zhou**

Supporting Information

Atomic-Scale Visualization and Quantification of Configurational Entropy in Relation to Thermal Conductivity: A *Proof-of-principle* Study in *t*-$GeSb_2Te_4$

Yongjin Chen, Bin Zhang, Yongsheng Zhang, Hong Wu, Kunling Peng, Hengquan Yang, Qing Zhang, Xiaopeng Liu, Yisheng Chai, Xu Lu, Guoyu Wang, Ze Zhang, Jian He, Xiaodong Han, Xiaoyuan Zhou**

Including:

1. The "nearest" neighbor atomic column intensity differential analysis (IDA) method
2. Structure models of *trigonal* $Ge_1Sb_2Te_4$ at different entropy levels
3. Simulated HAADF STEM images
4. The structure and the denotation of crystallographic directions of *t*-$Ge_1Sb_2Te_4$
5. Calculations of configurational entropy
6. Configurational entropy induced phonon scattering
7. The sound velocities and related mechanical properties
8. Density function theory calculations
9. Calculated thermal conductivity
10. Differential thermal analysis (DTA)
11. Hall mobility
12. Lorenz number and thermal diffusivity
13. The reproducibility and thermal cycling stability
14. Thermal transport properties between 5 K and 773 K
15. Electrical transport and thermoelectric performance
16. Supplementary references

1. The "nearest" neighbor atomic column intensity differential analysis (IDA) method

The high angle annular dark field scanning transmission electron microscopy (HAADF-STEM) image is chemical element discernable, the brightness is roughly proportional to the exponential function of the atomic number, aka the so-called Z contrast image. So far, there are mainly two methods both referred to as quantitative HAADF, library matching [1-3] and statistical decomposition [4].

Here we developed a "nearest" neighbor atomic column intensity differential analysis (IDA) method, which effectively reduces the undesirable influence of experimental noise and thickness. The IDA method utilizes the contrast of HAADF intensity to offer an unprecedented atomic-level microscopic picture of atomic structure. There are mainly several steps in the IDA quantification method: (1) re-scaling the image intensity by measuring the intensity of the incident electron probe but subtracting the vacuum intensity; (2) normalizing each atom column intensity by the nearest neighbor atomic column intensity differential analysis; (3) obtaining the site occupancy of atoms; (4) comparing the image intensities with simulated image intensities. Here we provided a model of atomic-scale visualization and quantification in single crystalline *trigonal* $GeSb_2Te_4$ (aka *t*-$GeSb_2Te_4$) with native atomic site disorder.

The imaging process is carried out by a piece of self-developed MATLAB code. The HAADF images are modelled as a combination of Gaussian peaks, which can be described as:

$$I\,(x,y) = I_0 + \sum_i A_i\, exp\left[\frac{(x-x_i)^2+(y-y_i)^2}{-2\omega_0^2}\right]\ , (1)$$

Where I_0 is the background intensity, A_i is the amplitude of each peak. All of the Gaussian peaks shared the same width ω_0. The experimental images are pixels × pixels, corresponding to nm×nm.

Theoretically, the incident probe intensity and the HAADF detector should be taken into account in the quantitative HAADF simulation process. [1-3] We measured the intensity of the incoming beam and obtained the electron probe on an absolute scale by applying the method of LeBeau *et al.* [1-3], but without external amplifier. **Figure S1** shows the color map of the intensity of the incident electron probe while scanning over the detector and the radially averaged sensitivity of the detector as a function of the scattering angle. And then the GST image intensities relative to the intensity of the incoming electron beam allowed us to direct compare with simulated image intensities. The absolute normalized intensity can be expressed as $I_{norm} = \frac{I_{raw}-I_{vac}}{I_{det}-I_{vac}}$, where I_{raw} is the raw intensity, I_{vac} is the intensity in the vacuum region, I_{det} is the averaged intensity on the detector.

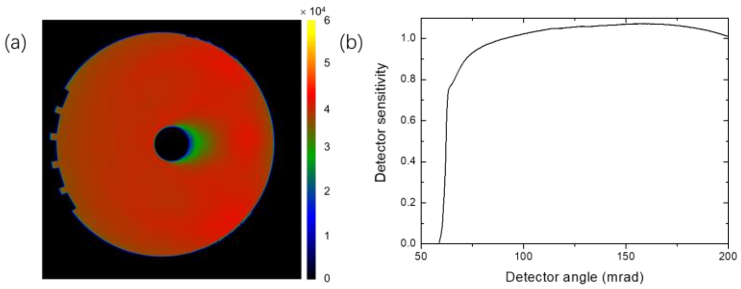

Figure S1 (a) Color map of the intensity of the incident electron probe while scanning over

the detector, (b) the radially averaged sensitivity of the detector as a function of the scattering angle.

In general, the crystal structure of $Ge_1Sb_2Te_4$ (lattice system: rhombohedral, crystal system: trigonal, space group$R\bar{3}m$) is derived from the cubic ABC stacking sequence along c axis consisting of three septuple layers interpenetrated by van der Waals gaps, which can be represented in terms of the stacking of the close-packed planes described by AβCαBγA BγAβCαB CαBγAβC. The capital Roman letters represent Te layers and the Greek letters represent the Ge/Sb layers. Thus, the stacking sequence of $Ge_1Sb_2Te_4$ is -Te1-GS2-Te2-GS1-Te2-GS2-Te1-, where Te atoms occupy two non-equivalent sites (denoted as Te1 and Te2), and similarly, GS1 and GS2 are non-equivalent octahedral cation sites for Ge and Sb atoms, thereby forming Te sublattice and Ge/Sb sublattice. The atomic number difference between Te and Ge/Sb naturally bring about the contrast in HAADF-STEM image. **Figure S2** shows the HAADF-STEM image of GST along the $[11\bar{2}0]$ direction, and the inset is the corresponding fast Fourier transform (FFT) image. The intensity fluctuations of Ge/Sb sublattice (the weak peak between two adjacent strong peaks from Te sublattices) in the intensity line-profile that correspond to the septuple layers marked by the red box indicate cation site occupancy disorder.

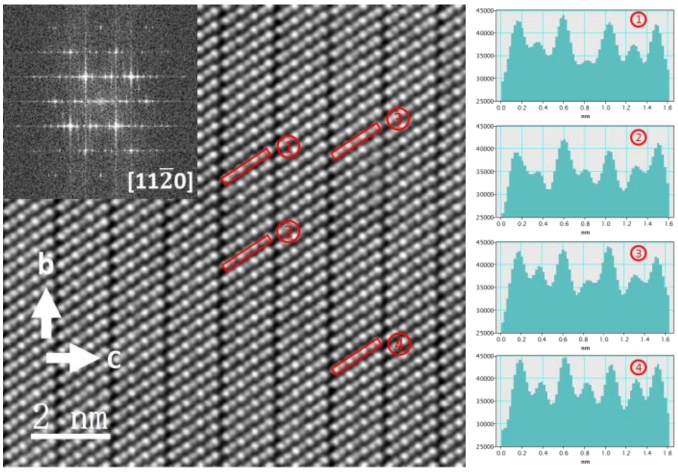

Figure S2 The filtered HAADF-STEM image of GST along the $[11\bar{2}0]$ direction, and the inset is the corresponding FFT pattern. The intensity line-profile (right panel) of septuple layers marked by the red boxes.

First, we obtain the absolute intensity and the position of each atomic column in a HAADF image based on the Gaussian distribution as shown in **Figure S3**.

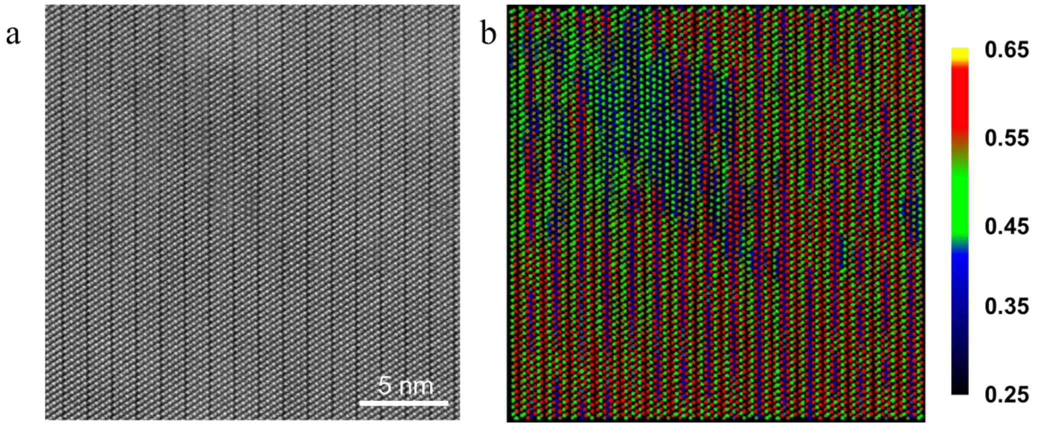

Figure S3 (a) The filtered HAADF-STEM image of GST along the $[11\bar{2}0]$ direction, and (b) the corresponding absolute intensity by normalizing the measured image intensities to the incident beam.

Then, we apply the IDA method to better visualize the change of spot brightness in the HAADF image. Specifically, the absolute intensity of each Te column in **Figure S3(b)** (cf. the solid circles in the middle in **Figure S4(b)**) is first divided by the average intensity of its "nearest" Te neighbors (cf the dotted circles in the same figure) as the normalized intensity of Te sublattice, the result is shown in **Figure S4(c)**. The intensity of a Ge/Sb column is further divided by the average intensity of adjacent Te columns (taken as the reference, cf. **Figure S4(b)**) to obtain the normalized intensity of Ge/Sb column, which indirectly reflects the cation atoms distribution in the columns as shown in **Figure S4(d)**. The IDA process can be described using the following two equations.

$$I_a = n * I_{a0} / \sum_1^n I_{Te} \quad , (2)$$

$$I_c = m * I_{c0} / \sum_1^m I_{Te} \quad , (3)$$

where the I_{Te} denotes the absolute intensity from the HAADF image of the neighboring Te columns (cf. the red circles in **Figure S4(b)**), I_{a0} and I_{c0} are the intensities of center anion Te and those of center cation Ge/Sb (cf. the yellow circles in **Figure S4(b)**), respectively. The I_a and I_c are the normalized intensity of anion column and cation column, respectively; n, m are the numbers of the neighboring Te columns for the center anion Te and cation Ge/Sb columns, respectively.

Specifically, the number of the neighboring Te columns for the center anion Te is six in GST, and the number of the neighboring Te columns of the center cation Ge/Sb columns is four. (*n*=*6*, *m*=*4*). So the normalized intensity equations of GST show as following:

$$I_a = 6 * I_{a0} / \sum_1^6 I_{Te} \quad , (4)$$

$$I_c = 4 * I_{c0} / \sum_1^4 I_{Te} \quad , (5)$$

The normalized intensities are represented by different colors (see the colorbar) in the normalized maps (**Figure S4(c) and (d)**). In IVV-V_2VI_3 pseudo-binary compounds, intrinsic (native) point defects refer to vacancies and antisite defects, the formation of which intimately depends on the stoichiometry [5-6]. Excess Te in the starting materials of synthesis facilitates the formation Ge and Sb vacancies on random cationic sites, whereas deficient Te would facilitate anion antisites. Indeed, the color gradient of Te_1 layers indicates a large amount of anionic antisites resulting from the off-stoichiometry (**Table S2**). Meanwhile, Sb_{Te}' and Ge_{Te}'' anionic antisites responsible for the observed high carrier concentration. We calculated the

defect concentration from the measured carrier concentration of 4.3 × 10^{20} cm^{-3} to be about 2.25% Sb_{Te} antisites defects or 1.13% Ge_{Te} antisites defects as tolerable and energy favorable (**Figure S18**). From the cation normalized map we conclude that the Ge is rich in the middle layer than the outer layers.

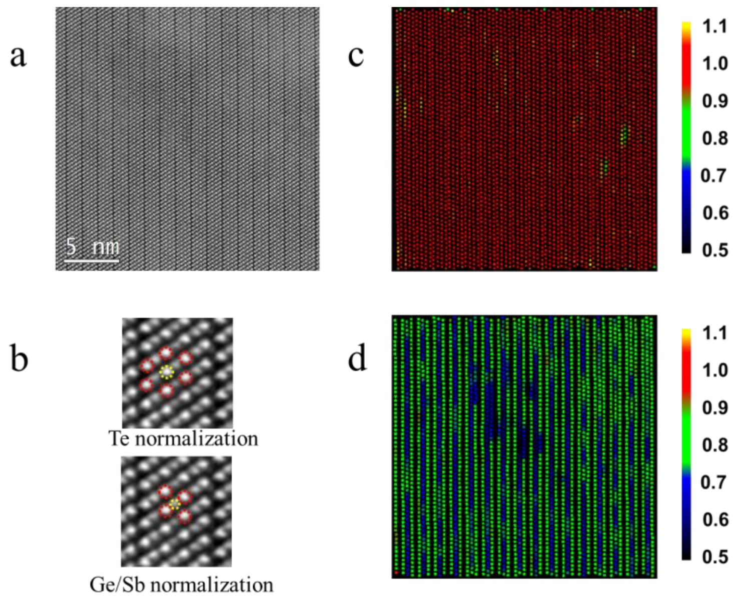

Figure S4 The schematic diagrams of the IDA method and the results. (a) A typical HAADF image of GST along $[11\bar{2}0]$ direction. (b) Simple sketches of IDA methods. (c, d) The normalized image for anion (Te) sublattice and cation (Ge/Sb) sublattice, respectively.

Beyond the normalized intensity, we further carry the quantitative analysis of the cation distribution down to the atomic level. As known, the brightness of the HAADF-STEM image is roughly proportional to the exponential function of the atomic number. Here we suppose $Ge_{occupancy} + Sb_{occupancy} = 1$, and the cation vacancy is left out due to the small amount. Then we calculate the quantitative results of the cation distribution using **Equation 6**:

$$\frac{a*Z_{Ge}^{\alpha}+(1-a)*Z_{Sb}^{\alpha}}{Z_{Te}^{\alpha}} = \frac{I_{cation}}{I_{Te}} \quad , (6)$$

where a is the Ge percentage in each cation column, I_{Ge}, I_{Sb} and I_{Te} are the intensity, z_{Ge}, z_{Sb} and z_{Te} are the atom mumber of Ge, Sb and Te, respectively. The I_{cation}/I_{Te} ratio is the normalized intensity value. The normalized intensities obey a Gaussian distribution, so the mean value is identified as 50% Ge-occupancy and 50% Sb-occupancy based on the literature.[7-12] The exponential α is found to be 1.74, which is different from the square of atomic numbers as commonly used. The scattering cross-sections are highly sensitive to collection angle [13], surface strain fields [14], the number of atoms in a column and the chemical composition [15], the real exponent will be lower than expected. The darkest atomic column corresponds to about 82.0% Ge-occupancy. In our IDA method, the ratio of the normalized intensity was used to calculate the quantitative results of the cation distribution as following: $\frac{I_{norm}^{A}}{I_{norm}^{B}} = \frac{I_{raw}^{A}-I_{vac}}{I_{raw}^{B}-I_{vac}}$. So, the intensity of incident electron probe did not influence the results in the quantitative process.

The curve of the Ge percentage as a function of normalized intensity is shown in **Figure S5**. The point of intersection with vertical axis represents the normalized intensity as 100% Ge occupancy. Similarly, the point of intersection with horizontal axis represents 100% Sb. Based on the normalized intensity value of each atom column, we can map the cation distribution (**Figure 1i** and **Figure 1j)** in the main text. The Ge occupancy of each atom column is thus derived. **Figure S6** shows the statistics data of atomic column Ge occupancy of GS1 and GS2 cationic sublattice sites. Finally, we obtain the quantitative analysis of the cation distribution to reach an unprecedented atomic level. The average model of our results *is* $-Te-Ge_{26.9}/Sb_{73.1}-Te-Ge_{49.9}/Sb_{50.1}-Te-Ge_{26.9}/Sb_{73.1}-Te-$, a statistical result over 7000 atoms columns. Likewise, we can quantify the anion distribution as well, assuming 2.25% Sb_{Te} or 1.13% Ge_{Te} antisites defects.

Atom type	Ge	Sb	Te
Z	32	51	52

Table S1 The atomic number of Ge, Sb and Te, respectively.

	Theory（%）	Experiment（%）
Ge	14.29	15.2
Sb	28.57	29.8
Te	57.14	55.0
Total	100	100

Table S2 The chemical content of the ideal $Ge_1Sb_2Te_4$, as well as the real single crystal GST measured by the super X-ray energy dispersive spectrometer (EDS).

Defects type	$Ge_{vacancy}$（%）	$Sb_{vacancy}$（%）	Sb'_{Te}（%）	Ge'_{Te}（%）
Concentrations	4.5	1.50	2.25	1.13

Table S3 The concentration of the defects when the carrier concentration reaches 4.3×10^{20} cm^{-3}.

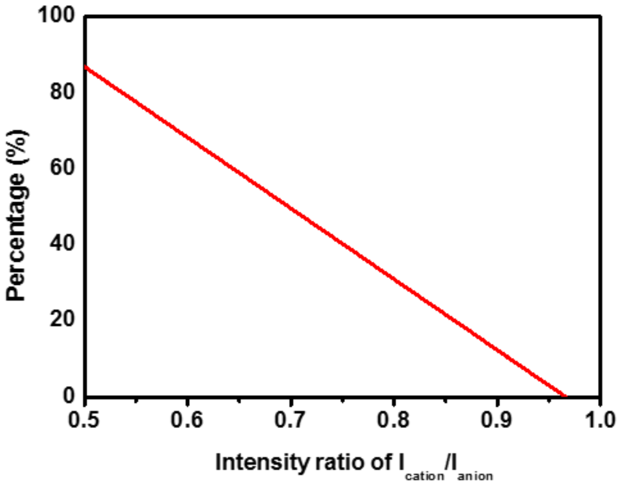

Figure S5 The Ge percentage in the cation (Ge/Sb) column as a function of normalized intensity.

	μ	sigma	FWHM
GS1	49.9	6.0	14.1
GS2	26.9	6.0	14.1

Table S4 The fitting values of Gauss distribution of both GS1 and GS2 cationic columns.

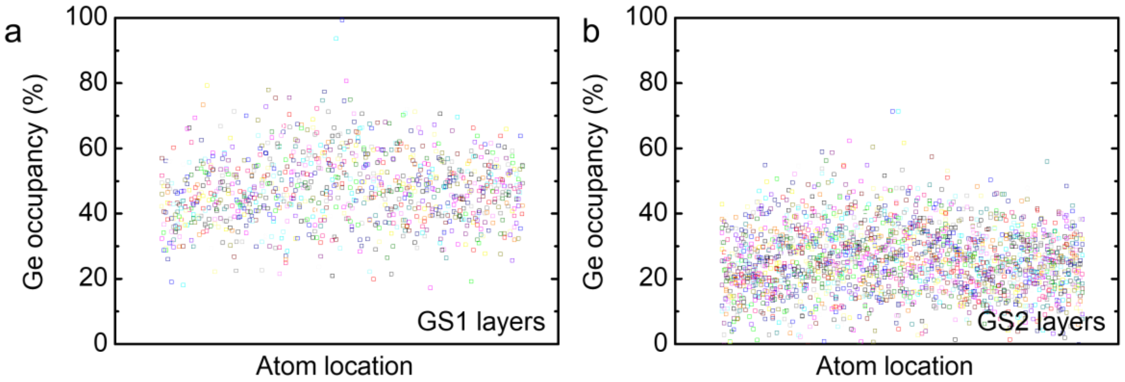

Figure S6 The statistics data of atomic column Ge occupancy of GS1 and GS2 cationic sublattice sites.

The source-of-error analysis the IDA method

The statistical histograms of Te normalized intensity over 4000 atom columns show that Te_2 obeys the Gaussian distribution while Te_1 deviate that in **Figure S7**. The full width at half maximum (FWHM) of the intensity distribution is composed of the intrinsic atom disorder distribution fluctuation and instrument resolution errors,

$$F(i, q) = D(i) \otimes E(q), (7)$$

where the symbol '⊗' represents convolution, F(i), D(i) and E(q) present the fluctuation sum, disorder part and instrument part, respectively. The instrument resolution error bar of TEM and IDA in quantification analysis is estimated to be about 4%, evaluated on the FWHM of the Te2 peak shown in **Figure S7**.

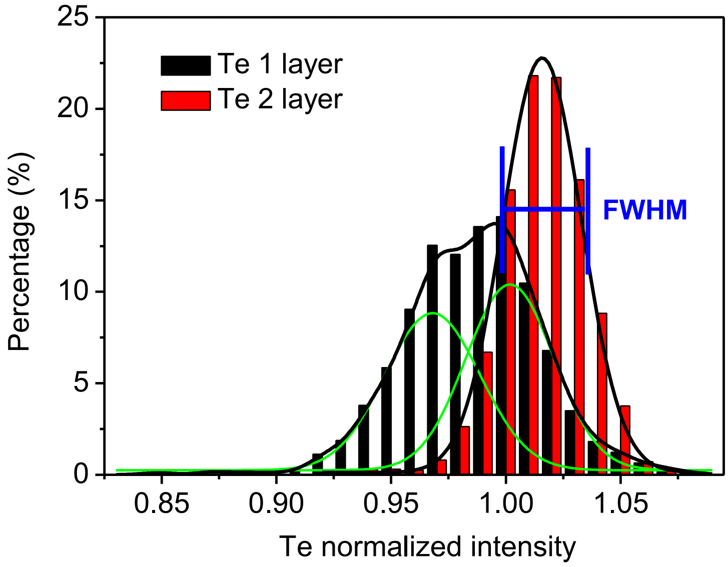

Figure S7 The statistical histogram of Te normalized intensity over 4000 atom columns. The error bar of TEM and IDA in quantification analysis, evaluated on the full width at half maximum (FWHM) of the Te2 peak, is estimated less than 4%.

2. Structure models of trigonal $Ge_1Sb_2Te_4$ at different entropy levels

The model of Agaev *et al.* [11] *-Te-Sb-Sb-Te-Te-Ge-Te-*
The model of Kooi *et al.* [7] *-Te-Sb-Te-Ge-Te-Sb-Te-*
The model of Sun *et al.* [8] *-Te-Sb-Te-Ge-Te-Sb-Te-* (density functional theory calculations)
The model of Matsunaga *et al.* [9] *-Te-Ge_{25}/Sb_{75}-Te-Ge_{50}/Sb_{50}-Te-Ge_{25}/Sb_{75}-Te-*
The model of Karpinsky *et al.* [10] *-Te-Ge_{26}/Sb_{72}-Te-Ge_{43}/Sb_{57}-Te-Ge_{26}/Sb_{72}-Te-*
The model of Lotnyk *et al.* [12] supports the results of Matsunaga *et al and* Karpinsky *et al*.

3. Simulated HAADF STEM images

HAADF imaging gives highly intuitive images and can often be directly interpreted on a semi-quantitative level (*Z-contrast images*), however, the combined elastic and thermal diffuse scattering (TDS) make the background somewhat complicated, especially when we try to extract the quantitative information of the sample at the atomic scale. [16] Image simulations are applied for a comparison of experiment and simulation on the same absolute intensity scale with atomic column resolution. The effects of dynamical scattering of the probe and also the contribution of thermal diffuse scattering must be included in the simulation. Meanwhile, the effects of channeling can be significant, because atomic resolution HAADF STEM is often performed on crystals aligned along high-symmetry directions where the atoms form resolvable columns. [16] So far, the approaches to including both channeling and TDS in the calculations fall into two main categories: those that use absorptive potentials and those that make use of the frozen phonon approach.

In this section, some of the detailed simulation results are presented, which used the Dr. Probe® simulation software [17], providing quantitative HAADF-STEM image simulations at the atomic scale. Five atomic structure models are input for the simulations, example images are displayed in **Figure S8** for different thicknesses of a $GeSb_2Te_4$ crystal in $[11\bar{2}0]$

orientation. The simulation is done for an aberration-free 300 keV electron probe with 25 mrad semi-convergence angle and an effective source size of 80 pm. Defocus is 0.00 nm, and detectors are placed on the optical axis in the diffraction plane as a HAADF detector from 80 mrad to 200 mrad. The sample thicknesses in the simulation vary from 0.4272 nm to 54.68 nm (from 1 to 129 slices), and we select the thickness about 5 nm, 10 nm, 15 nm, 20 nm, 25 nm, 30 nm, 35 nm, 40 nm, 45 nm, 50 nm and 55nm (corresponding to 12, 24, 36, 48, 60, 72, 84, 96, 108, 120, 129 slices) shown in **Figure S8.** The HAADF images have obvious difference in the different models, and gradually change with thickness. The sample thickness and Ge/Sb site disorder have an effect on the intensity.

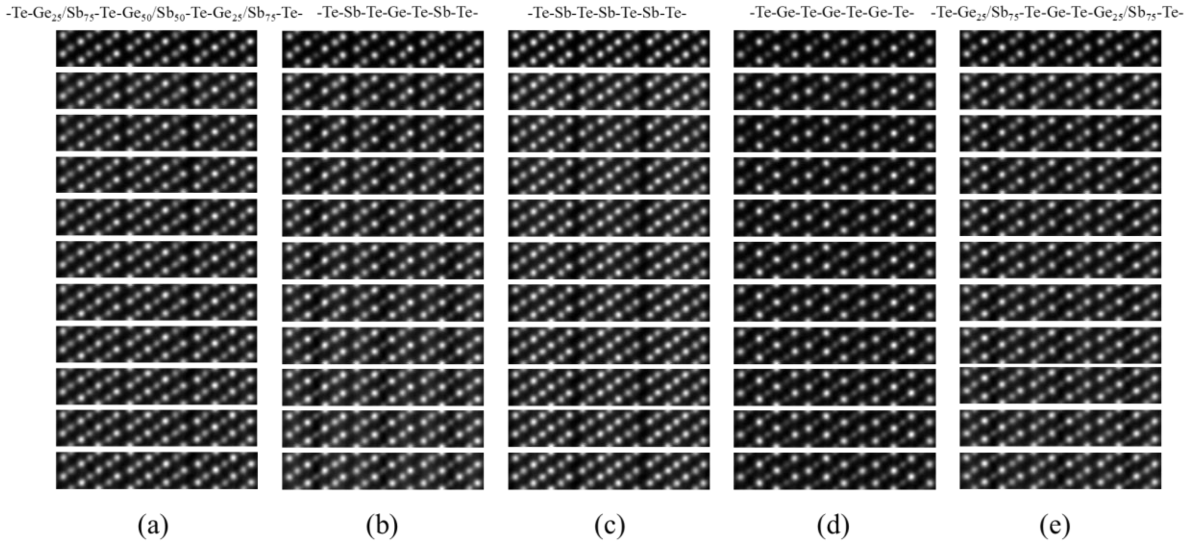

Figure S8 HAADF-STEM image simulations for different thicknesses of five atomic structure models of $GeSb_2Te_4$ crystal in $[11\bar{2}0]$ orientation at the atomic scale by Dr. Probe. (a) -Te-Ge_{25}/Sb_{75}-Te-Ge_{50}/Sb_{50}-Te-Ge_{25}/Sb_{75}-Te-; (b) -Te-Sb-Te-Ge-Te-Sb-Te-; (c) -Te-Sb-Te-Sb-Te-Sb-Te-; (d) -Te-Ge-Te-Ge-Te-Ge-Te-; (e) -Te-Ge_{25}/Sb_{75}-Te-Ge-Te-Ge_{25}/Sb_{75}-Te-.

Through careful analysis, **Figure S9** shows a comparison of experiment and simulation on the same absolute intensity scale with atomic column resolution. We randomly pick and extract the intensities of three septuple-structures using the IDA method, the results agree with the Matsunaga model (-Te-Ge_{25}/Sb_{75}-Te-Ge_{50}/Sb_{50}-Te-Ge_{25}/Sb_{75}-Te-) (cf. the first section of Supporting Materials, the “nearest” neighbor atomic column intensity differential analysis).

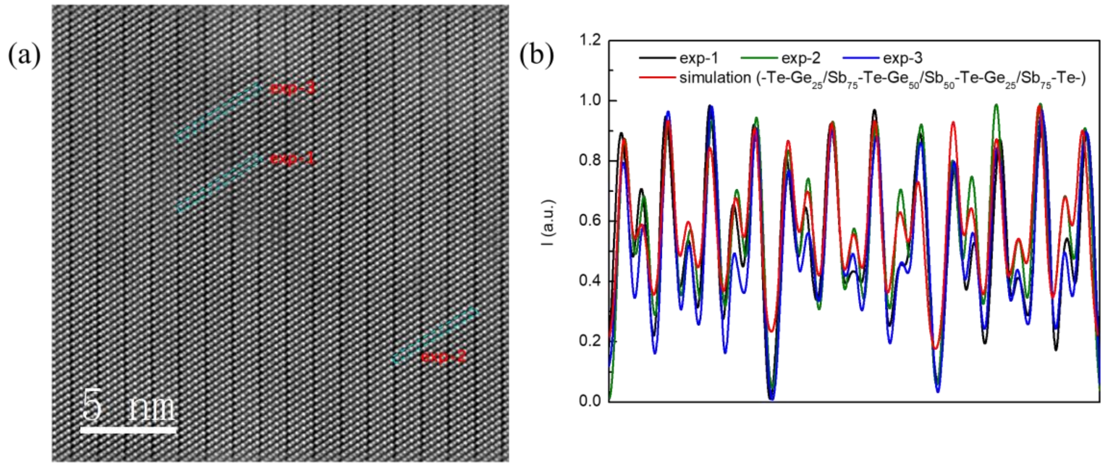

Figure S9 Comparison of experiment and simulation on the same absolute intensity scale with atomic column resolution.

Besides, we analyze the exponent α of simulation images by IDA. HAADF imaging gives highly intuitive images and can often be directly interpreted on a qualitative level (*Z-contrast images*). The intensity of scattering would follow the Rutherford scattering model of being proportional to Z^2 where Z is the atomic number of the illuminated atom. The Rutherford scattering model assumes an unscreened Coulomb potential, but in practice screening will reduce the Z-dependence to some extent. Simulations of scattering intensities using realistic atomic scattering factors suggest that a slightly-lower-than-2 exponent (1.6-1.9) is more reasonable for thin samples. Due to the effects of dynamical scattering of the probe, the channeling effect (Atomic resolution HAADF STEM is often performed on crystals aligned along high symmetry directions where the atoms form resolvable columns), thermal diffuse scattering (TDS) and sample thickness, the real exponent will be much lower than expected.[18,19] We randomly pick and extract the intensities of three septuple-structures using the IDA method, the results agree with the Matsunaga model (-Te-Ge_{25}/Sb_{75}-Te-Ge_{50}/Sb_{50}-Te-Ge_{25}/Sb_{75}-Te-) (cf. the first section of Supplementary Materials, the "nearest" neighbor atomic column intensity differential analysis). Besides, we also analyze the exponent α of simulation images by IDA. In our simulation of $GeSb_2Te_4$, the exponent α of simulated images can be as high as about 1.98 for thin samples, and as low as about 1.89 for thicker sample (about 50 nm), which is very close to the value used in our IDA result (1.74). Above all, the IDA method proposed in **Section 1** allows us to statistically extract and quantify the site disorder of HAADF-STEM images, which are comparable with the reference of multislice simulations given by Dr. Probe ®software.

4. The structure and the denotation of crystallographic directions of *t*-$Ge_1Sb_2Te_4$.

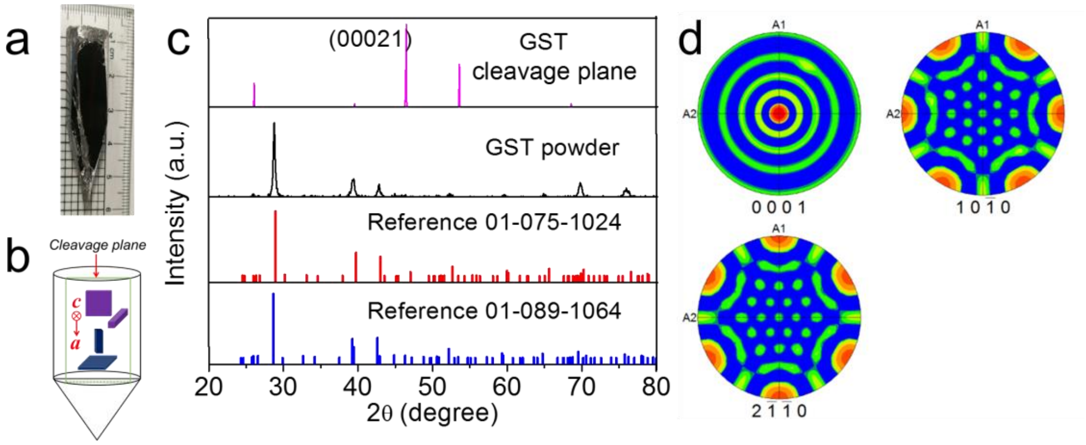

Figure S10 (a) A typical crystal cleaved along the (0001) plane. (b) The cartoon illustration of specimens cutting along the two specified axes（*a* and *c*）for transport measurements. (c) XRD measurements of the GST single crystal on the cleavage plane and GST powder, and the reference diffraction patterns ($Ge_{0.95}Sb_{2.01}Te_4$ and $Ge_1Sb_2Te_4$). The (00021) reflection plane indicates that the GST crystal is cleaved at the plane that is perpendicular to *c*-axis. (d) The electron backscatter diffraction (EBSD) analysis, the pole figures of samples were used as an assistant to confirm *a*-axis.

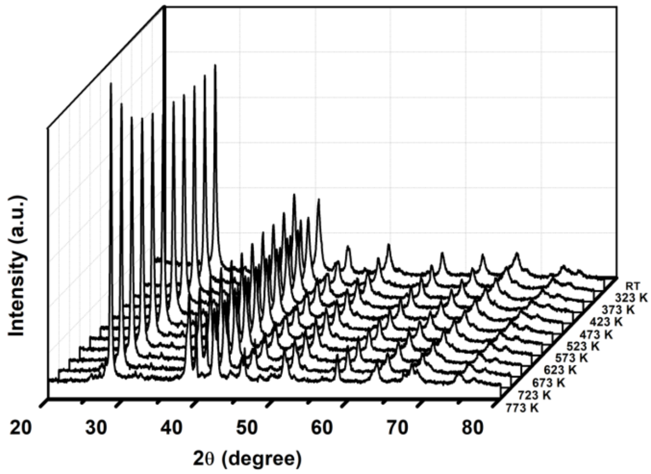

Figure S11 XRD measurement of GST at different temperature (from room temperature to 773 K) using PANalytical Empryean with CuKα radiation.

5. Calculations of configurational entropy

The Boltzmann's formula of configurational entropy stemming from the occupation of any microstate is assumed to be equally probable for an isolated system at a global thermodynamic equilibrium, [20] which is in contrast to our findings that Ge/Sb presents Gauss distribution in microscopic scale as shown in **Figure 1** (cf. main text). The data of atomic site disorder presented in the preceding section allow us to statistically extract and quantify the configurational entropy. Here, we define the configurational entropy of ith atomic column as S_i based on the concept of entropy and expressed in terms of a discrete set of probabilities [21]

$$S_i = -\sum_j^n f_{ij} \log_b f_{ij}, \quad \sum_j^n f_{ij} = 1 \, , (8)$$

where f_{ij} is the mole content of the jth component of ith atomic column probability function, b is the base of the logarithm. Here the value of b is Euler's number e, and the corresponding units of entropy are the *nats*. Entropy is extensive in nature, so the macroscopic configurational entropy is a sum over all the atomic columns. The entropy per mole is $S = N_A S_i$, in the unit of J K^{-1} mol^{-1}. For two different atoms on each sublattice, f_1 and f_2 are the fractional occupancies, $f_1 + f_2 = 1$. The expression of **Equation 8** will be

$$S_i = -[f_1 lnf_1 + f_2 lnf_2] = -[f_1 lnf_1 + (1-f_1)ln(1-f_1)] \text{ , (9)}$$

We define $P = f_1 lnf_1 + (1-f_1)ln(1-f_1)$, and the Taylor expansion of $H(f) = f$ at $f = 1$ is

$$\ln(1+f) = f - \frac{f^2}{2} + o(f^3) \ldots , -1 < f < \infty \text{ , (10)}$$

$$P = f_1 lnf_1 + (1-f_1)ln(1-f_1) = f_1 \ln(1+f_1-1) + (1-f_1)ln(1-f_1) \approx f_1 \left[(f_1 - 1) - \frac{(f_1-1)^2}{2}\right] + (1-f_1)\left[-f_1 - \frac{(-f_1)^2}{2}\right] = -2.5 f_1(1-f_1) = -2.5 f_1 f_2 \text{ , (11)}$$

We can now substitute **Equation 11** into **Equation 9** to calculate the configurational entropy.

$$S_i = 2.5 * f_1 f_2 \text{ , (12)}$$

For *t-GST*, the occupancies of Ge/Sb in the crystallographic sublattices are approximate normal distribution, $\psi(\mu,\delta) = \frac{1}{\sqrt{2\pi\delta^2}} e^{-\frac{(f-\mu)^2}{2\delta^2}}$, where μ and δ are the expectation and variance, respectively, 0<*f*<1, its configurational entropy will become

$$S_{av} = \int_0^1 \frac{2.5f(1-f)}{\sqrt{2\pi\delta^2}^2} e^{-\frac{(f-\mu)^2}{2\delta^2}} df \approx \int_{-\infty}^{+\infty} \frac{2.5f(1-f)}{\sqrt{2\pi\delta^2}} e^{-\frac{(f-\mu)^2}{2\delta^2}} df = 2.5[\mu(1-\mu) - \delta^2], 0<f<1 \text{ , (13)}$$

The statistical histograms of configurational entropy of *t-GST*

A wave-like vibration mode of the CE along the *a*-axis of GS1 and GS2 lattices are revealed. The pair correlation function *p(r)* can be used to describe the fluctuation period. [22] The discrete data points of GS1 layers and GS2 layers are used to calculate the pair correlation function by the self-developed MATLAB code, the defined correlation function can be expressed as $p(r) = \frac{1}{N}\sum_{r<j-i<r+\Delta r} S_i S_j$, where N is the total number of pairs of S_i and S_j. $S_{i,j}$ present the configurational entropy values at the distance range of r, where r is the spacing between any two CE peaks in a particular atomic layer. The smooth curves of GS1 layers reveal a high frequency CE modulation (1/3 Å^{-1}) and the short-range order of Ge/Sb, and the characteristic length of modulation of atomic column CE of GS1 layers is estimated to be close to the interatomic spacing ~3 Å. By contrast, a sharp distinctive feature occurs in the GS2 layers, and the characteristic length for GS2 layers are found to be at about 9 Å with a low frequency CE modulation (1/9 Å^{-1}). Extended to the data containing 60 × 50 (layers) atom columns, the CE of GS1 layers and GS2 layers are shown in **Figure S12**, reflecting the fluctuation features of CE. GS1 layers are in a status of high CE values, with most GS1 layers are beyond 0.6 *nats* and the amplitude *h* about 0.05 *nats*, which correspond to an atomic ratio of Ge/Sb ~ 1:1. In contrast, the entropy values of Sb-rich GS2 layers are mostly between 0.4 *nats* and 0.69 *nats* with the amplitude *h* about 0.15 *nats*. The average entropy value of GS1 layers is 0.68 *nats* and 0.57 *nats* for GS2 layers, and the S_{av} of *t-GST* cationic sublattice is 0.61 *nats*. The amplitude h of configurational entropy modulation can be estimated in the sense of

unbiased minimum variance.

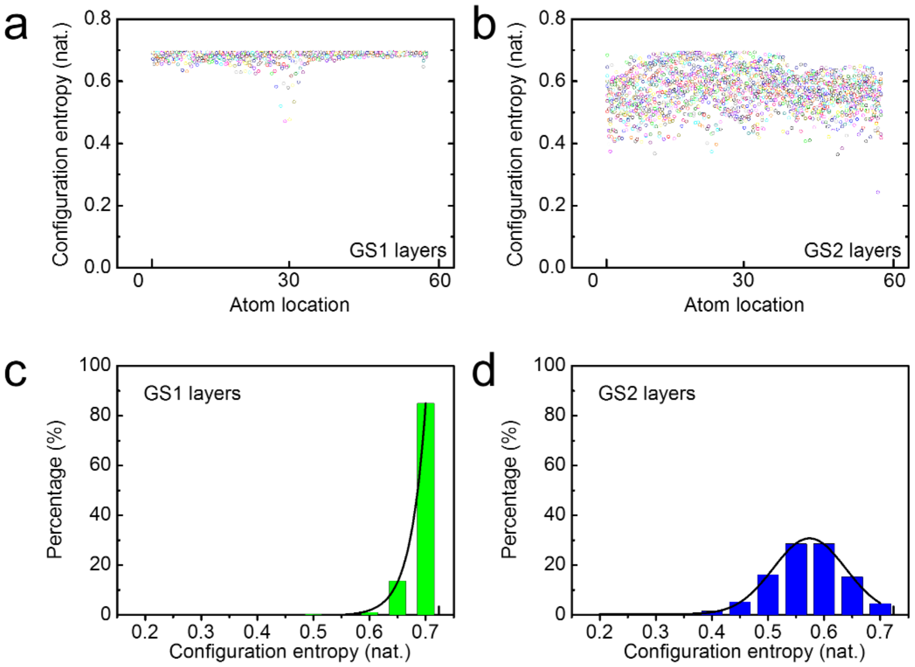

Figure S12 The entropy statistical histograms of GS1 and GS2 cationic sublattice sites over 3000 cation columns.

6. Configurational entropy induced phonon scattering

The disorder scattering parameter Γ, where the scattering parameters Γ_M and Γ_S are due to mass and strain field fluctuations, respectively.

$$\Gamma = \Gamma_M + \Gamma_S \ , (14)$$

The chemical composition of a material can be expressed as $A_{1c1}A_{2}c_{2}A_{3c3}A_{4c4}...A_{ncn}$, where the A_i are crystallographic sublattices in the structure and the c_i are the relative degeneracies of the respective sites. In general, there will be several different types of atoms that occupy each sublattice, and the kth atom of the ith sublattice has mass M_i^k, radius r_i^k, and fractional occupation f_i^k.

The average mass and radius of atoms on the ith sublattice are:

$$\bar{M}_i = \sum_k f_i^k M_i^k \ , (15)$$

$$\bar{r}_i = \sum_k f_i^k r_i^k \ , (16)$$

The average atomic mass of the compound is:

$$\bar{\bar{M}} = \frac{\sum_{i=1}^{n} ci\bar{M}_i}{\sum_{i=1}^{n} ci} \ , (17)$$

The mass fluctuation scattering parameter is then given by:

$$\Gamma_M = \frac{\sum_{i=1}^{n}(\frac{\bar{M}_i}{\bar{\bar{M}}})^2\Gamma_M^i}{\sum_{i=1}^{n} ci} \ , (18)$$

where the mass fluctuation scattering parameter for the ith sublattice is:

$$\Gamma_M^i = \sum_k f_i^k(1-\frac{M_i^k}{\bar{M}_i})^2 \ , (19)$$

For two different atoms on each of the ith sublattices, i.e., $k=1, 2$. In the case of *t-GST*, n=2 because of 2 sublattices. We have masses M_i^1 and M_i^2, fractional concentrations f_i^1 and

f_i^2, and $f_i^1 + f_i^2 = 1$ and $f_i^1 M_i^1 + f_i^2 M_i^2 = \overline{M}_i$, **Equation 18** becomes

$$\Gamma_M = \frac{\sum_{i=1}^{n} ci(\frac{\overline{M}_i}{\overline{\overline{M}}})^2 f_i^1 f_i^2 (\frac{M_i^1 - M_i^2}{\overline{M}_i})^2}{\sum_{i=1}^{n} ci} \text{ , (20)}$$

The strain field fluctuation scattering parameter is then given by:

$$\Gamma_S = \frac{\sum_{i=1}^{n} ci(\frac{\overline{M}_i}{\overline{\overline{M}}})^2 f_i^1 f_i^2 \varepsilon_i (\frac{r_i^1 - r_i^2}{\bar{r}_i})^2}{\sum_{i=1}^{n} ci} \text{ , (21)}$$

Where $f_i^1 r_i^1 + f_i^2 r_i^2 = \bar{r}_i$, and ε_i is a phenomenological adjustable parameter for the ith sublattice. The parameter ε_i is a function of the Grüneisen parameter γ, which characterizes the anharmonicity of the lattice.

7. The sound velocities and related mechanical properties [23-28]

Sound velocity is another important parameter to understand the thermal transport in a solid. The measured sound velocities and series calculation values present in **Table S5**. The longitudinal (v_l), transverse (v_t), and mean (v_m) sound velocities for the GST along c axis are 2700 m s^{-1}, 1773 m s^{-1} and 1941 m s^{-1}, respectively. The phonon mean-free path of GST is estimated to be 0.36 nm according the measured sound velocities. The experimental κ_{L-exp} values are close to the amorphous limit (κ_{Lmin}) estimated by the Cahill approximation model [29].

Measurements of sound velocities are carried out on the pellet samples at room temperature. Longitudinal and transverse sound velocities are measured using pulse-receiver (®Olympus-NDT) equipped with an oscilloscope (®Keysight). Shear gel (®Olympus) and water are used as couplants between the sample and the ultrasonic transducers for transverse and longitudinal sound velocity measurements, respectively.

Average sound velocity (ϑ_m) is calculated from the longitudinal sound velocity (ϑ_l) and the transverse sound velocity (ϑ_t).

$$\frac{1}{\vartheta_m} = \sqrt[3]{\frac{1}{3}(\frac{1}{\vartheta_l^3} + \frac{2}{\vartheta_t^3})} \text{ , (22)}$$

Young's modulus (E) is calculated by

$$E = \frac{\rho \vartheta_t^2 (3\vartheta_l^2 - 4\vartheta_t^2)}{(\vartheta_l^2 - \vartheta_t^2)} \text{ , (23)}$$

where ρ is the sample density.

Poisson ratio (ϑ_p) is calculated by

$$\vartheta_p = \frac{1 - 2(\vartheta_t/\vartheta_l)^2}{2 - 2(\vartheta_t/\vartheta_l)^2} \text{ , (24)}$$

Shear modulus (G) is calculated by

$$G = \frac{E}{2(1 + \vartheta_p)} \text{ , (25)}$$

Phonon mean free path (l) is calculated by

$$\kappa_L = \frac{1}{3} C_V \vartheta_m l \text{ , (26)}$$

where C_V is the heat capacity at constant volume.

Direction	ϑ_l (m/s)	ϑ_t (m/s)	ϑ_m (m/s)	E (GPa)	G (GPa)	ν_p	l (nm)	κ_{L-exp} (W/m·K) (T=300K)	κ_{L-min} (W/m·K)
a	4660	3018	3311	129.7	56.9	0.139	0.96	1.4	0.59
c	2700	1773	1941	44.8	20.0	0.121	0.30	0.4	0.29

Table S5 The sound velocities (longitudinal sound velocity ϑ_l and the transverse sound velocity ϑ_t) and the experimental lattice thermal conductivity (κ_{L-exp}) of two directions of GST. The Mean sound velocity (ϑ_m), Young's modulus (E), Shear modulus (G), Poisson ratio (ν_p) and Phonon mean free path (l) are derived by **Equation 22-26** based on the measured sound velocities. The theory lowest lattice thermal conductivity (κ_{L-min}) are calculated by the Cahill approximation model [24].

8. Density function theory calculations

Structure models

Here we compare two systems in terms their thermodynamic stability. (1) The pristine ordered GST: Ge ions occupy the GS1 layer while Sb ions occupy the GS2 layer. The other system is disordered GST, there is cross-layer site occupational disorder, sometimes called 'self-solid-solution'. The disordered GST is simulated using a Special Quasi-random Structure (SQS) [29] and the Alloy Theoretic Automated Toolkit (ATAT) [31], which satisfies the mixing ratios of Ge/Sb, (2) Ge : Sb = 1 : 1 at the GS1 layer, and Ge : Sb = 1 : 3 at the GS2 layer, which is a high entropy state.

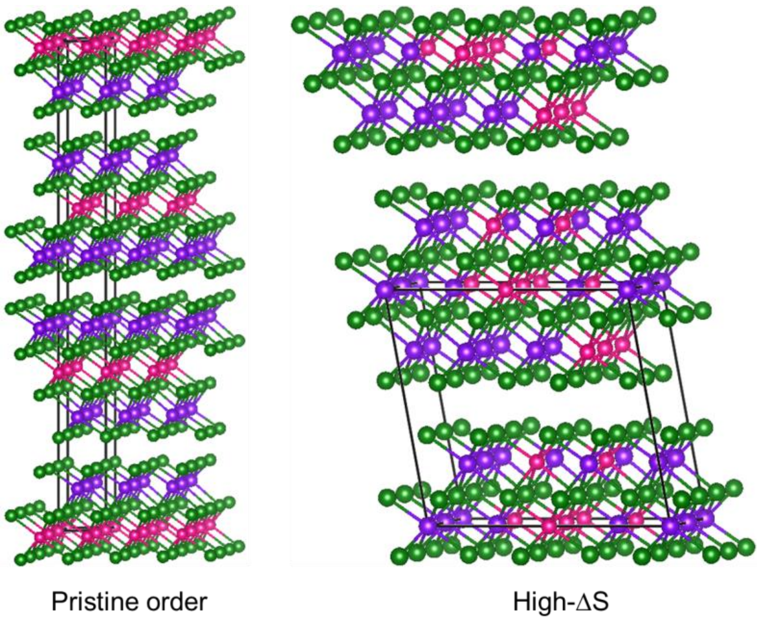

Figure S13 The structure models of GST in the pristine order and high entropy disorder system, Te (cyan), Sb (purple), Ge (pink).

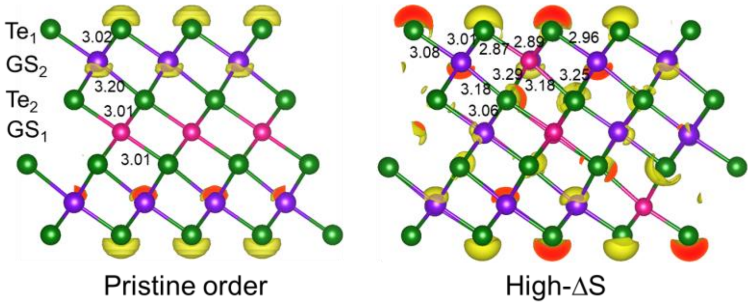

Figure S14 The electron localization function (ELF) and bonding lengths of GST in the pristine order and high entropy disorder system.

Since the Ge-Sb-Te system is a layered structure and contains heavy elements (Sb and Te), we need to test the effects of van der Waals (VdW) interactions and spin-orbital (SO) coupling on the energetic trend. The VdW corrections is used the Tkatchenko-Scheffler (TS) method [32]. We calculate the energy differences of the two compounds (pristine order and high entropy) with the VdW, SO or VdW+SO corrections, and compare them with the simple PBE results (**Figure S15**). It turns out both the stability trend and the energy differences using the corrections are similar to those using PBE. This is due to that the three Ge-Sb-Te compounds share the same layered structure and the same elements. This confirms that the PBE calculations are suitable to investigate the phase stability trend of the two compounds.

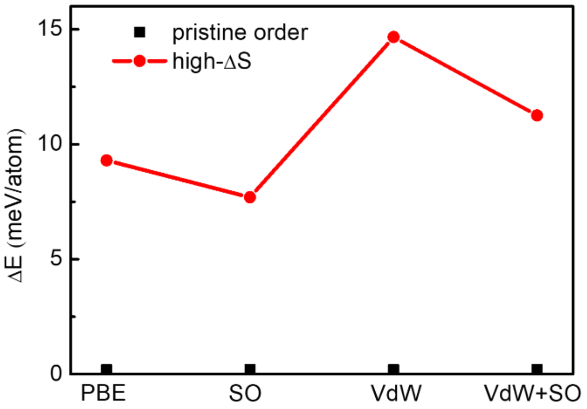

Figure S15 Theoretically calculated energy differences of the pristine order and high-entropy Ge-Sb-Te compounds using the PBE functional, SO, VdW and VdW+SO corrections.

The geometry structure parameters (aka the lattice constants and interlayer distances) of the pristine $GeSb_2Te_4$ using the PBE functional and the VdW corrections are presented in Table S6. We find that the in-plane lattice constant is the same with or without the VdW corrections. While along the *c* direction, the VdW corrections shrink the interlayer distance and the lattice constant along *c* axis. Smaller interlayer distance implies stronger interlayer interactions and thus higher lattice thermal conductivity along the *c* direction. Nevertheless, we notice that the pristine and high entropy configurations undergo similar trend of the interlayer distance change after including the VdW corrections (Table S6). Therefore, whether including VdW corrections or not won't alter our conclusions on the lattice thermal conductivity.

Structure $GeSb_2Te_4$	Geometry	PBE	PBE+VdW
Pristine (R-3m)	Lattice constants<br><br><br>Interlayer distance	a=4.308 Å<br>c=41.974 Å<br>γ=120°<br>3.13 Å	a=4.297 Å<br>c=40.570 Å<br>γ=120°<br>2.78 Å
High-S (P1)	Lattice constants<br><br><br><br><br><br>Interlayer distance	a=14.537 Å<br>b=8.588 Å<br>c=14.904 Å<br>α=90.02°<br>β=99.72°<br>γ=89.98°<br>3.35 Å	a=13.882 Å<br>b=8.578 Å<br>c=14.872 Å<br>α=90.02°<br>β=100.12°<br>β=89.98°<br>2.85 Å

Table S6 The geometry structures of the pristine and high-S $GeSb_2Te_4$ compounds using the PBE functional and VdW corrections.

We use the Deybe model to evaluate the vibrational properties [33,34]. The enthalpy (H_{vib}) and vibrational entropy (S_{vib}) can be written as,

$$H_{vib} = \int_0^{\omega_D} d\omega (\frac{V\omega^2}{2\pi^2 v^3})(\frac{\hbar\omega}{e^{\hbar\omega/k_BT} - 1}) \quad , (27)$$

$$S_{vib} = k_B \int_0^{\omega_D} d\omega \left(\frac{V\omega^2}{2\pi^2 v^3}\right) (\frac{\hbar\omega/k_BT}{e^{\hbar\omega/k_BT} - 1} - ln(1 - e^{-\hbar\omega/k_BT})) \quad , (28)$$

where, ω, v, $\hbar$ and k_B are the frequency, phonon velocity, Boltzmann constant, and Plank constant, respectively. ω_D is the Debye frequency ($\omega_D = \Theta k_B/\hbar$, Θ is the Debye temperature). Further including the configurational entropy (S_{conf}), we can evaluate the Gibbs free energy as formula

$$G = E + H_{vib} - TS_{vib} - TS_{conf} \quad , (29)$$

The configurational entropy plays a role of stabilizing the *t-GST* phase by theoretical calculations of Gibbs free energy in **Figure 4c** of main text.

The electronic band structure

The calculated band gap of pristine order model and high entropy model are ~ 0.33 eV and ~ 0.14 eV, respectively.

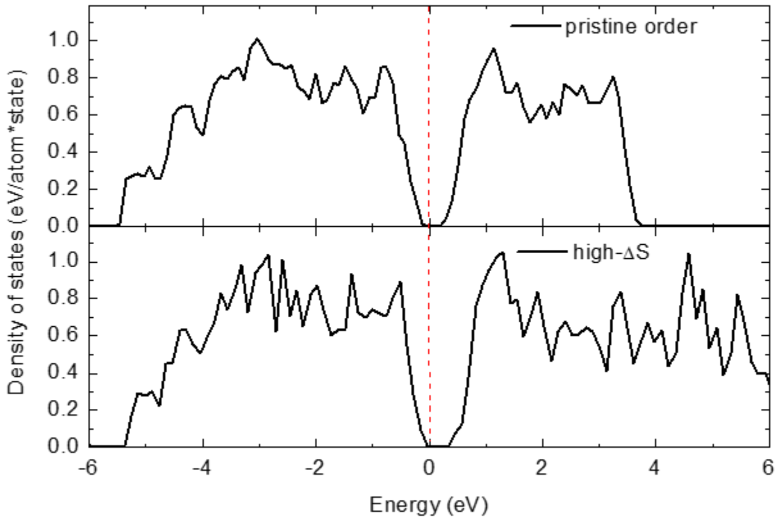

Figure S16 The calculated density of states of the pristine order and high entropy models of GST.

Formation energy of various point defects

In $Ge_1Sb_2Te_4$ alloys, inherent point defects occur during the crystal growth from the stoichiometric melt. A large amount of anion antisite defects result from the off-stoichiometric feature (**Table S2**). Negatively charged Sb_{Te}' and Ge_{Te}'' anion antisite defects is reasonable upon the deficiency of Te atoms, which accounts for the p-type conductivity. The smaller the differences in electronegativity and atomic size between cation and anion atoms, the less resistance to the formation of antisite defects. [35]

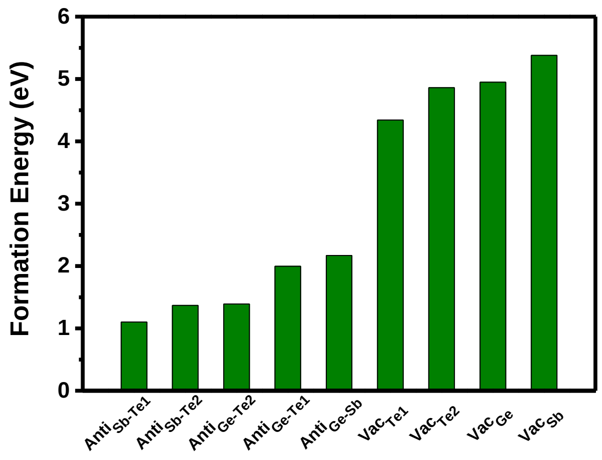

Figure S17 The formation energy of various point defects calculated by the density functional theory (DFT), which indicates the formation of anion antisite defects and cation disorder is more thermodynamically favorable than the vacancy in GST.

Lattice distortion effects

The lattice distortion of the local octahedron motif of $(Ge)Te_6$ in the disorder GST system is more severe than that in order GST. The details of bonding lengths and bonding angles present in **Figure S17**.

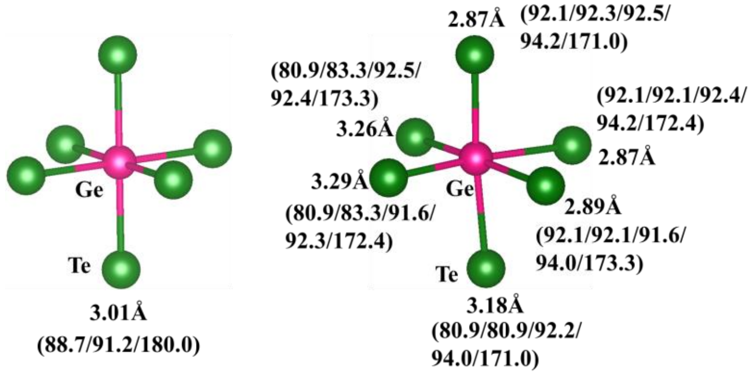

Figure S18 The bonding lengths and bonding angles of the local octahedron motif of $(Ge)Te_6$

in the pristine order and high entropy GST system.

Anharmonicity

Since the Grüneisen parameter characters the lattice anharmonicity (the relationship between phonon frequency and crystal volume change), low Young's modulus, shear modulus, and large Gruneisen parameter usually imply a low lattice thermal conductivity. **Table S7** shows the calculated bulk and shear moduli (B and G in GPa), phonon velocities (v in m/s), Grüneisen parameters (γ), and acoustic Debye temperatures (Θ_D in K) of both pristine order and high entropy GST systems. The calculated results show that the high entropy (GST-SQS) has lower phonon velocity and larger Grüneisen parameter than that of the pristine order structures.

$Ge_1Sb_2Te_4$	E_g (eV)	B (GPa)	G (GPa)	ν_L (m/s)	ν_S (m/s)	ν (m/s)	γ_L	γ_S	γ	Θ_D (K)
pristine order	0.33	25	23	3000	1933	2122	2.5	1.8	2.0	73
disorder (high entropy)	0.14	17	10	2235	1264	1406	3.6	3.0	3.2	34

Table S7 Calculated band gaps (eV), bulk and shear moduli (B and G in GPa), phonon velocities (v in m/s), Grüneisen parameters (γ), and acoustic Debye temperatures (Θ_D in K) of pristine order and high entropy $GeSb_2Te_4$ systems.

9. The thermal conductivity calculation

The relaxation time of the cationic location disorder scattering. [36,37]

$$\tau_S^{-1} = \frac{\omega^4 \bar{V}}{4\pi v^3}(\Gamma_M + \Gamma_S) \ , (30)$$

Where ω is the phonon frequency, $\bar{V}$ is the average volume per atom, v is the acoustic phonon velocity, Γ_M is the mass fluctuation and and Γ_S is the strain fluctuation due to the Ge/Sb disorder.

The relaxation time of the cationic location disorder scattering only considering the mass fluctuation is given by [37]:

$$\tau_M^{-1} = \frac{k_B^4 \bar{V} T^4}{4\pi \hbar^4 v^3} x^4 \sum_i f_i \left(\frac{\bar{m}-m_i}{\bar{m}}\right)^2 \ , (31)$$

Where k_B is the Boltzmann constant, $\hbar$ is the Plank constant, T is the absolute temperature, $x = \hbar\omega/k_B T$, m_i is the mass of the doping atom, f_i is the atomic occupation ratio, and $\bar{m}$ is the average mass of the unit cell.

The r-$GeSb_2Te_4$ has high site occupational disorder and thus high configurational entropy. It is challenging to derive the 2nd and 3rd order force constants required for the ShengBTE® code [38], not to mention the computation cost of computing anisotropic transport matrices. The Debye-Callaway model has drawbacks, e.g., the negligence of optical modes, may result in deviations of the absolute magnitude of lattice thermal conductivity. Nonetheless, acoustic modes are of utmost importance in the thermal transport. The efficacy of the Debye-Callaway

model has been proved in numerous studies, e.g., Cu-Sb-Se [39], SnSe [40] and a layered structure $Ge_2Sb_2Te_5$[41]. The Debye-Callaway model herein adopted include the effect of mass fluctuation (cf. **Eq. 4** in main text and **Eq. 31** in SI). For the purpose of unraveling the relation between entropy and lattice thermal conductivity in *r*-$GeSb_2Te_4$ with high native site disorder, the Debye-Callaway model correctly explains the trend of variation of lattice thermal conductivity and gives semi-quantitative yet microscopic insights, compared to other existing models.

We additionally provide the geometry structures (lattice constants and interlayer distances) of the pristine $GeSb_2Te_4$ using the PBE functional and the VdW corrections (Table S6). We find that the lattice constant in plane is the same with or without the VdW corrections. While along the c direction, the VdW corrections shrink the c axis and the interlayer distance. The smaller interlayer distance might indicate the stronger interatomic interactions and higher lattice thermal conductivity. Nevertheless, we notice that the three compounds (pristine and high-S) show the similar trend of the interlayer distance change with including the VdW corrections (Table S6). Therefore, this obviously will not change our conclusions on the lattice thermal conductivity.

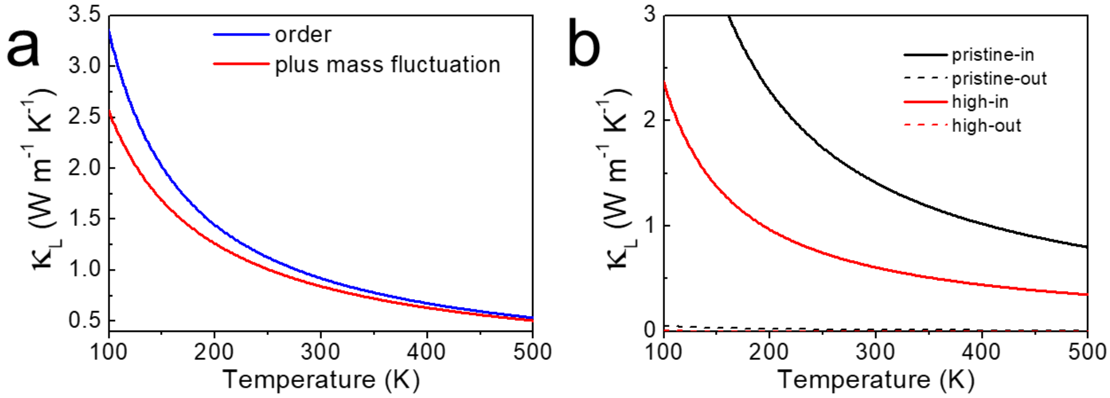

Figure S19 The calculated lattice thermal conductivity of order and disordered $GeSb_2Te_4$ using the elastic properties. (a) The red line presents the lattice thermal conductivity of disordered $GeSb_2Te_4$ only considering the ideal mass fluctuation. (b) The lattice thermal conductivity of pristine order and high entropy state of $GeSb_2Te_4$.

10. Differential thermal analysis (DTA)

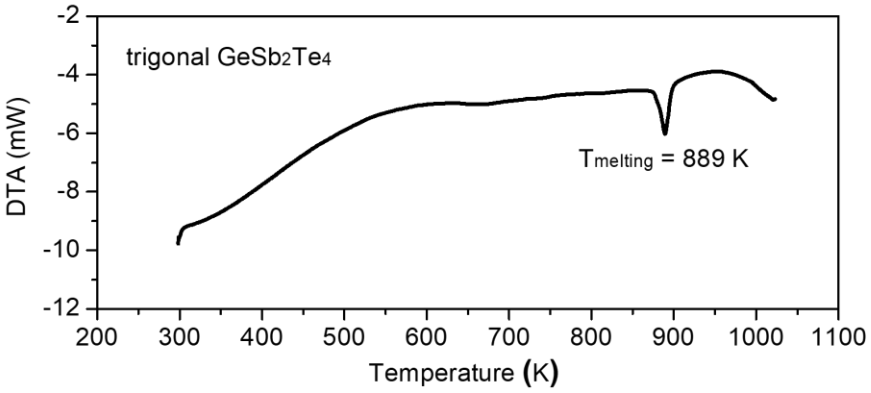

Figure S20 Differential thermal analysis (DTA) results indicate that GST appears to melt at 889 K with one endothermic peak observed on the heating curve.

11. Hall mobility

The carrier concentration is high at a level of about 4.3×10^{20} cm^{-3} at room temperature, which is relevant to the large density of native point defects. The Hall mobility exhibits an exponent behavior with temperature. A relatively weak temperature dependence of mobility presents at low temperature, which may be relative to configurational entropy. And acoustic phonon scattering is the dominant at higher temperatures.

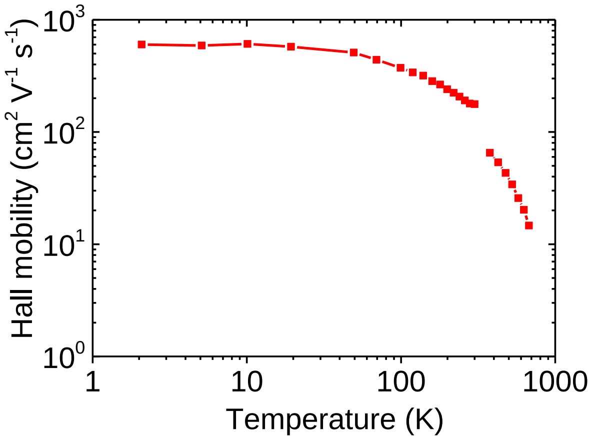

Figure S21 Hall mobility as a function of temperature for GST single crystals along *a-axis*.

12. Lorenz number and thermal diffusivity

For the $Ge_1Sb_2Te_4$ sample, the Lorenz number is calculated based on a single-band Kane model (SKB) [42,43] for the high carrier concentration, and we assume the charge carriers are mainly scattered by acoustic phonons.

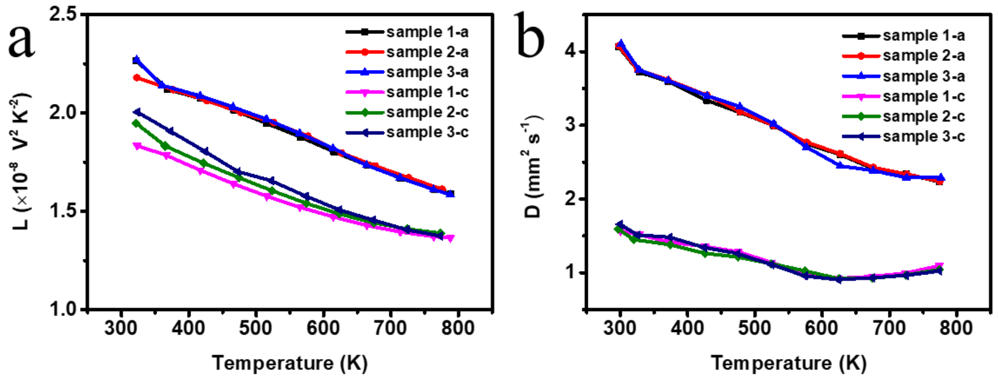

Figure S22 (a) The calculated Lorenz number derived from a single-band Kane model (SKB). (b) Thermal diffusivity as a function of temperature for GST single crystals along different directions.

13. The reproducibility and thermal cycling stability

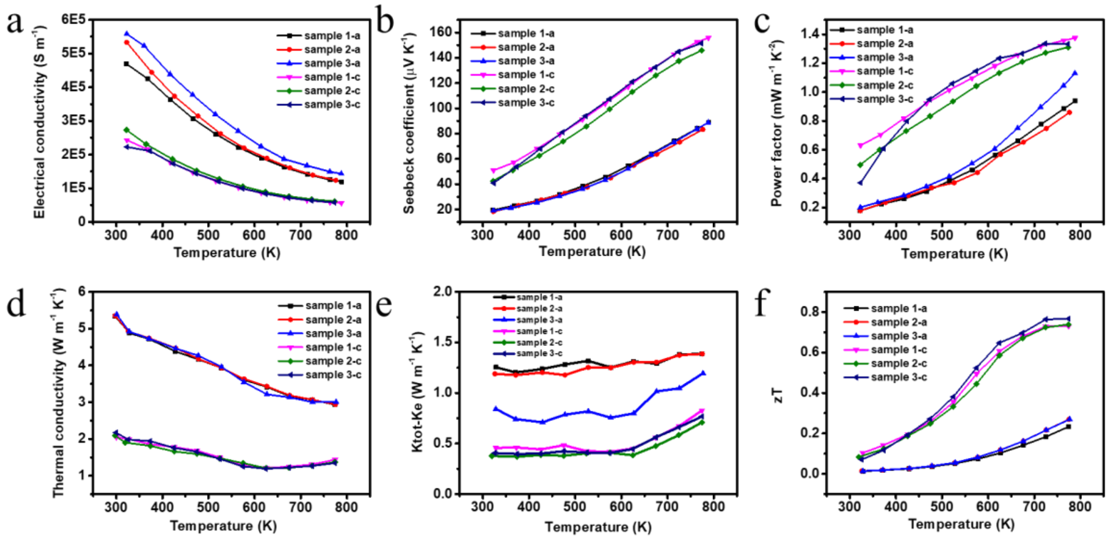

Figure S23 Reproducibility and thermoelectric properties as a function of temperature, for three samples of GST crystals along *a* and *c* axes. (a) Electrical conductivity. (b) Seebeck coefficient. (c) Power factor. (d) Total thermal conductivity. (e) The part of thermal conductivity. (f) ZT values.

14. Thermal transport properties between 5K and 773K

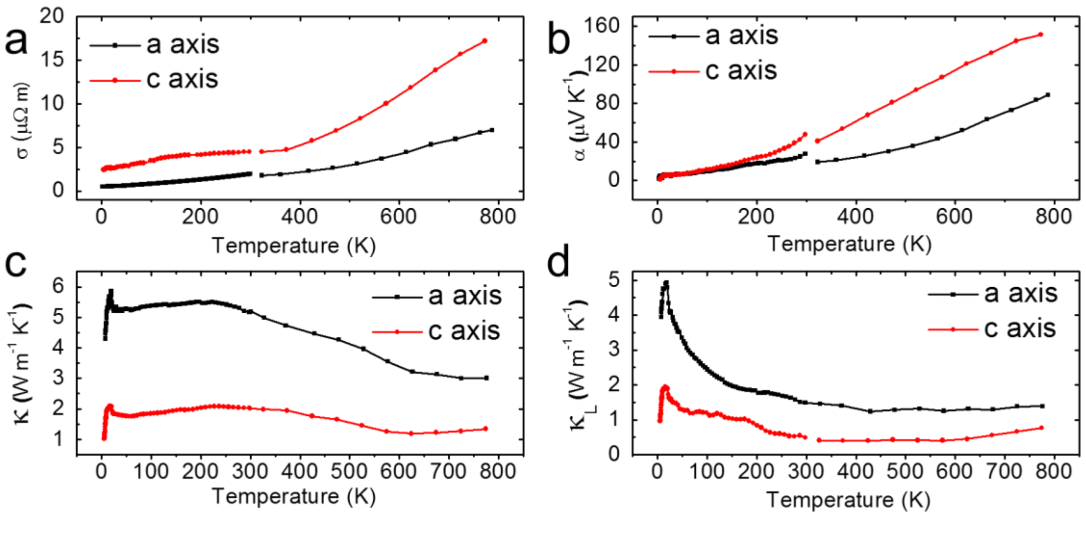

Figure S24 Thermoelectric properties as a function of temperature for GST crystals. (a) Electrical resistivity (σ). (b) Seebeck coefficient (α). (c) Total thermal conductivity (κ_{tol}). (d) Lattice thermal conductivity (κ_L).

A room temperature κ_L as low as 0.4 W m^{-1} K^{-1} is attained along *c*-axis of value of *t-GST*, comparable to other state-of-the-art single crystalline thermoelectric materials that are claimed to have ultralow thermal conductivity, e.g. SnSe[40], SnS[44,45] and $SnSe_{0.9}S_{0.1}$ [43](cf. **Fig. S25** in SI).

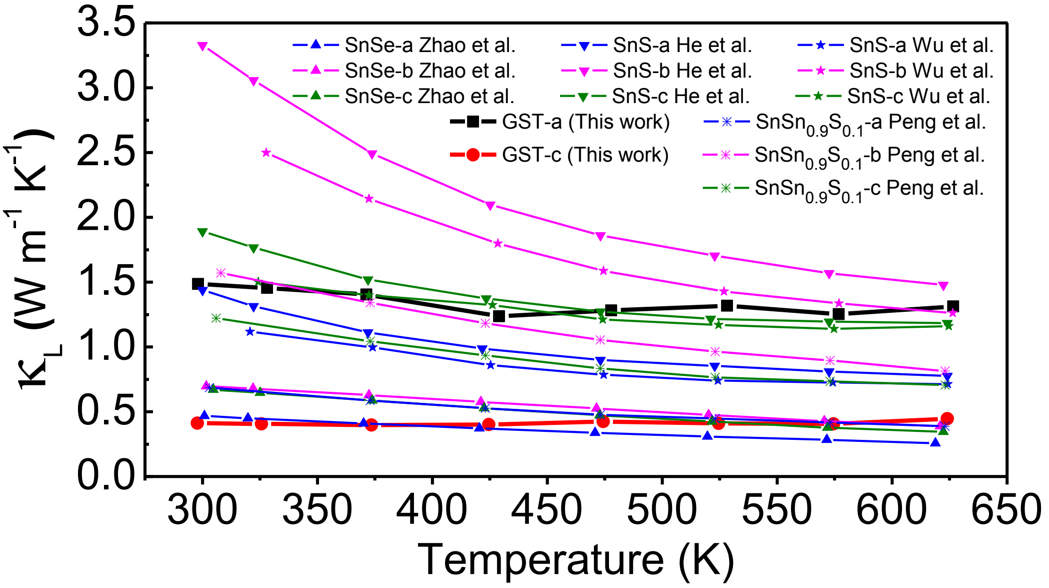

Figure S25 The lattice thermal conductivity (κ_L) of GST compared with SnSe, SnS and $SnSe_{0.9}S_{0.1}$ thermoelectric single crystals.

We compare the lattice thermal conductivity of *t-GST*, Sb_2Te_3[46,47] and GeTe in **Figure S26** and **Figure S27**, respectively. It is found that the lattice thermal conductivity of *t-GST* is lower than that of Sb_2Te_3, which had a sharp peak in temperature dependence. However, the peaks of *t-GST* are flatter, illustrating the significant influence of configurational entropy on thermal conductivity.

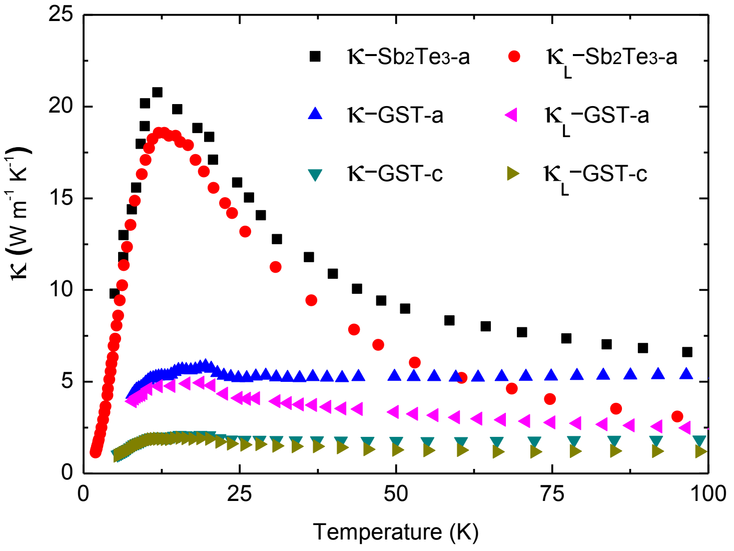

Figure S26 The total thermal conductivity and lattice thermal conductivity for Sb_2Te_3[46,47] and GST single crystals.

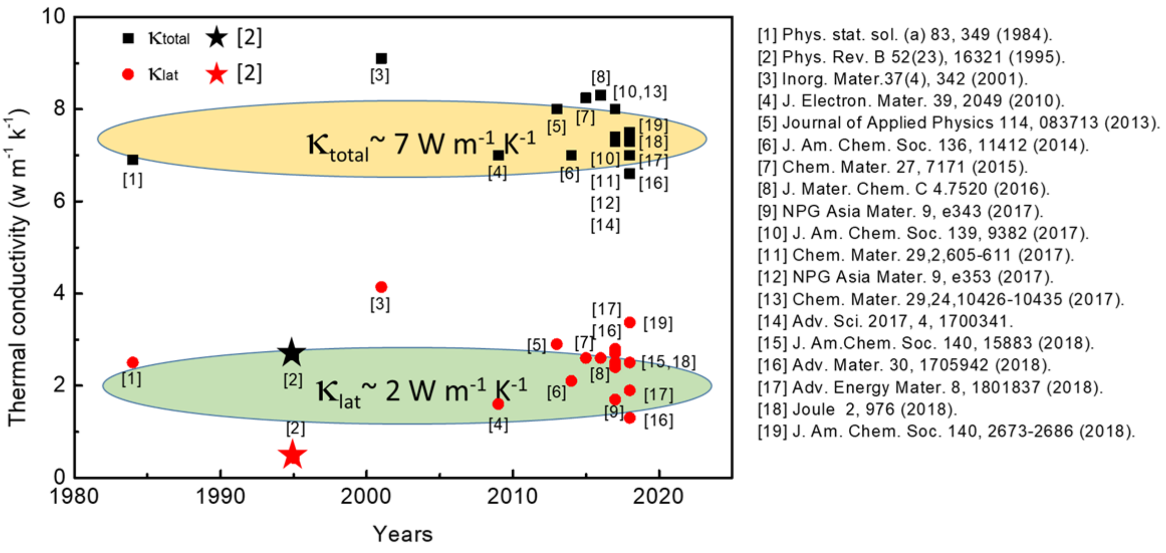

Figure S27 The reported available data of thermal conductivities of GeTe in the literature of the past decade. The solid black squares and red squares represent the total thermal conductivity and lattice thermal conductivity, respectively.

15. Electrical transport and thermoelectric performance

In general, entropy is intimately related to thermoelectrics: a thermoelectric process is a coupled process between charge and entropy flow (including heat flow and entropy creation); the nature of Seebeck coefficient is the average entropy carried per each charge carrier. The performance of thermoelectric materials can be expressed by the dimensionless figure of merit $zT=\alpha^2\sigma T/\kappa$, where α, σ, T and κ are the Seebeck coefficient, the electrical conductivity, the absolute temperature and the total thermal conductivity, respectively. In this section, we present the electrical transport and thermoelectric performance of single crystal *t-GST* in details, as shown in **Figure S28**. Electrical conductivities (~10^5 S m^{-1}) show a decrease tendency with rising temperature in **Figure S28a**, suggesting a degenerate semiconductor behavior, and σ along *a*-axis is almost two times higher than that along *c*-axis. The maximum *S* value reaches up to 151.5 μV K^{-1} at 773 K along *c*-axis in **Figure S28b**, because only high energy holes could pass over the energy barrier induced by *van der Waals* gaps. The strong anisotropy in electrical conductivity and Seebeck coefficient is probably due to the difference in carrier mobility along two directions, which is analogous to V_2VI_3 compounds [48]. The bipolar effect does not explicitly appear in the curves of temperature-dependent σ and α due to the high hole concentration. The power factor ($\alpha^2\sigma$) is displayed in **Figure S28c**. The highest power factor up to 1.34 mW $m^{-1}K^{-2}$ is observed along *c*-axis at 723 K, which is higher than that along *a*-axis (1.1 mW $m^{-1}K^{-2}$ at 773 K). The *zT* value as high as 0.8 at 723 K could be achieved along *c*-axis in **Figure S28d**, which is 8 times larger than the previously reported value of polycrystalline GST samples. [49,50] We also compare the thermal conductivity and thermoelectric *zT* of our single crystals *t-GST* with literature [50-52] in **Figure S29**. These results should be of interest to explore new TE materials in IVV-V_2VI_3 pseudo-binary compounds, such as $Ge_2Sb_2Te_5$, $Sn_1Sb_2Te_4$, $Sn_1Bi_2Te_4$ and $Pb_1Bi_2Te_4$ *et al*.

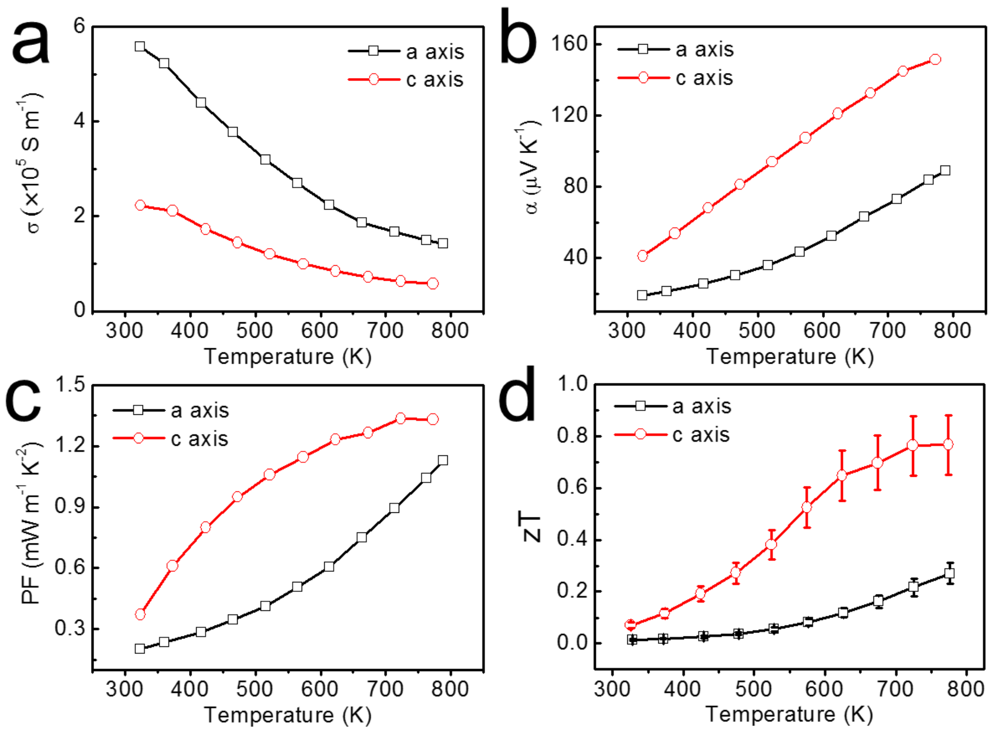

Figure S28. Thermoelectric properties as a function of temperature for *t-GST* crystals. (a) Electrical conductivity (σ). (b) Seebeck coefficient (α). (c) Power factor (PF). (d) Figure of merit (zT).

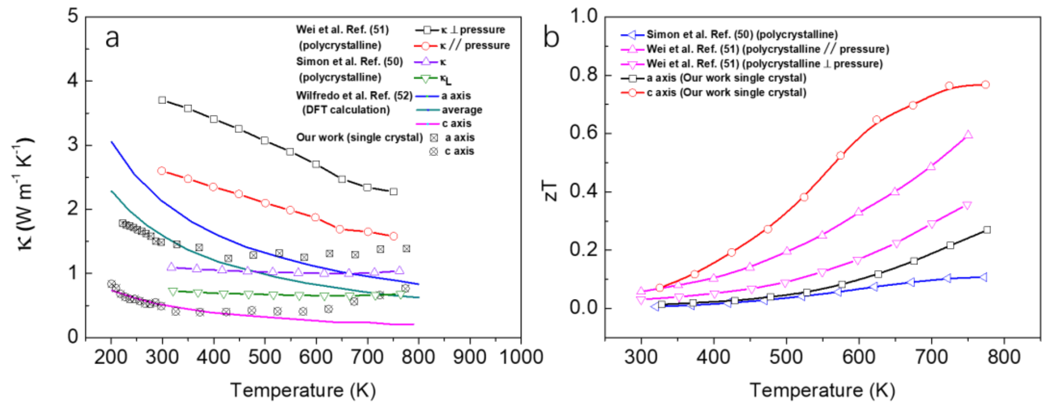

Figure S29. Comparison of the thermal conductivity (a) and thermoelectric performance zT (b) as a function of temperature for *t-GST* crystals. [50-52]

16. Supplementary references

[1] J. M. LeBeau, S. D. Findlay, L. J. Allen, S. Stemmer, *Phys. Rev. Lett.* **2008**, *100*, 206101.

[2] J. M. LeBeau, S. Stemmer, *Ultramicroscopy* **2008** *108*, 12.

[3] A. Rosenauera, K. Griesa, K. Mullera, A. Pretorius, M. Schowaltera, A. Avramescub, K. Englb, S. Lutgenb *Ultramicroscopy* **2009** *109*, 1171.

[4] S. Van Aert, A. De Backer, G. T. Martinez, B. Goris, S. Bals, and G. Van Tendeloo, *Phys. Rev. B* **2013** *87*, 064107.

[5] L. E. Shelimova OGK, P. P. Konstantinov, E. S. Avilov, M. A. Kretova, V. S. Zemskov, *Inorg. Mater.* **2004**, *40*, 451.

[6] P. P. Konstantinov, L. E. Shelimova, E. S. Avilov, M. A. Kretova, V. S. Zemskov, *Inorg. Maters.* **2001**, *37*, 662.

[7] B. J. Kooi, J. Th. M. De Hosson, *J. Appl. Phys.* **2002**, *92*, 3584.

[8] Z. M. Sun, S. Kyrsta, D. Music, R. Ahuja, J. M. Schneider, *Solid State Commun* **2007**, *143*, 240.

[9] T. Matsunaga, N. Yamada, *Phys. Rev. B* **2004**, *69*, 104111.

[10] O. G. Karpinsky, L. E. Shelimova, M. A. Kretova, J. P. Fleurial, *J. Alloy Compd.* **1998**, *268*, 112.

[11] K. Agaev, *Kristallogr* **1966**, *11*, 454.

[12] A. Lotnyk, U. Ross, S. Bernutz, E. Thelander, B. Rauschenbach, *Sci. Rep.* **2016**, *6*, 26724.

[13] S. Kim, Y. Jung, J. J. Kim, S. Lee, H. Lee, *J. Alloy. Compd.* **2015**, *618*, 545.

[14] T. Grieb T, *Ultramicroscopy* **2013**, *129*, 1.

[15] G. T. Martinez, van den Bos KHW, M. Alania, P. D. Nellist, S. Van Aert, *Ultramicroscopy* **2018**, *187*, 84.

[16] R. Brydson, *Aberration-Corrected Analytical Electron Microscopy*. Wiley, Hoboken, NJ, USA **2011**.

[17] J. Barthel, *Ultramicroscopy* **2018**, *193*, 1.

[18] P. Hartel, H. Rose, C. Dinges, *Ultramicroscopy* **1996**, *63*, 93.

[19] N. Tanaka, *Scanning Transmission Electron Microscopy of Nanomaterials: Basic of Imaging and Analysis*. Imperial College Press, London, GB **2015**.

[20] P. Perrot, *A to Z of Thermodynamics*. Oxford University Press, Oxford, NY, USA **1998**.

[21] C. E. Shannon, *Bell System Technical Journal* **1948**, *27*, 379.

[22] B. L. Gyorffy, G. M. Stocks, *Phys. Rev. Lett.* **1983**, *50*, 374.

[23] D. S. Sanditov, V. N. Belomestnykh, J. *Technical Phys.* **2011**, *56*, 1619.

[24] K. Kurosaki, A. Kosuga, H. Muta, M. Uno, S. Yamanaka, *Appl. Phys. Lett.* **2005**, *87*, 061919.

[25] B. Jiang, *Chem. Commun.* **2017**, *53*, 11658.

[26] Y. L. Pei, *NPG Asia Mater.* **2013**, *5*, e47.

[27] C. Wan, Z. Qu, Y. He, D. Luan, W. Pan, *Phys. Rev. Lett.* **2008**, *101*, 085901.

[28] J. Y. Cho, *Phys. Rev. B* **2011**, *84*, 085207.

[29] D. G. Cahill, S. K. Watson, R. O. Pohl, *Phys. Rev. B* **1992**, *46*, 6131.

[30] A. Zunger, S. Wei, L. G. Ferreira, J. E. Bernard, *Phys. Rev. Lett.* **1990**, *65*, 353.

[31] A. Van De Walle, *Calphad-computer Coupling of Phase Diagrams and Thermochemistry* **2013**, *42*, 13.

[32] M. S. Alexandre Tkatchenko, *Phys. Rev. Lett.* **2009**, *102*, 073005.

[33] C. Kittel, *Introduction to Solid State Physics*, Wiley, Hoboken, NJ, USA **2005**.

[34] Y. L.Wang, Y. S. Zhang, C. Wolverton, *Phys. Rev. B* **2013**, *88*, 024119.

[35] L. Hu, T. Zhu, X. Liu, X. Zhao, *Adv. Func. Mater.* **2014**, *24*, 5211.

[36] D. Wu, *Nano Energy* **2017**, *35*, 321.

[37] B. Abeles, *Phys. Rev.* **1963**, *131*, 1906.

[38] W. Li et al. *Comp. Phys. Commun.* **2014**, 185, 1747-1758.

[39] Y. Zhang, *Phys. Rev. B* **2012**, 85, 054306.

[40] L. Zhao et al. *Nature* **2014**, 508, 373-377.

[41] D. Campi, L. Paulatto, G. Fugallo, F. Mauri, M. Bernasconi, *Phys. Rev. B* **2017**, 95, 024311.

[42] Y. Pei, A. D. LaLonde, H. Wang, G. J. Snyder, *Energ. Environ. Sci.* **2012**, *5*, 7963.

[43] K. Peng *et al. Mater. Today.* **2017**, 21, 501-507.

[44] W. He *et al. J. Mater. Chem. A* **2018**, 6, 10048-10056.

[45] H.Wu *et al. Adv. Energy Mater.* **2018**, 8, 1800087.

[46] C. Drasar, M. Steinhart, P. Lost'ak, H. K. Shin, J. S. Dyck, C. Uher, *J. Solid. State. Chem.* **2005**, *178*, 1301.

[47] P. Lošt’ák, *J. Phys. Chem. Solids* **2006**, *67*, 1457.

[48] J. H. Dennis, *Adv. Energy. Conv.* **1961**, *1*, 99.

[49] S. Welzmiller, *Z. Anorg. Allg. Chem.* **2015**, *641*, 2350.

[50] S. Welzmiller, *Adv. Electron. Mater.* **2015**, *1*, 1500266.

[51] T. Wei, *Appl. Phys. Lett.* **2019**, 114, 053903.

[52] W. Ibarra-Hernández, *Phys. Rev. B* **2018**, 97, 245205.
